# Influence mechanisms of built environment factors on residential electricity carbon emissions under carbon peaking pressure: a case study of Wuqing District, Tianjin, China

**DOI:** 10.3389/fpubh.2026.1859904

**Published:** 2026-09-07

**Authors:** Feng Chaoxian, Zhang Xiaoping, Cui Zixuan

**Affiliations:** School of Architecture and Urban Planning, Shandong Jianzhu University, Jinan, China

**Keywords:** advanced metering infrastructure (AMI), built environment factors (BEF), influence mechanism, residential electricity carbon emissions, Wuqing District, Tianjin

## Abstract

In light of the “dual carbon” objectives, examining residential electricity carbon emissions (RECE) is crucial for urban low-carbon advancement. This research investigates 146 micro-scale residential zones in Wuqing District, Tianjin, emphasizing the influence of building environmental factors (BEF) on RECE. This study innovatively employs AMI smart meter data from 2021 to 2023 to establish a framework of “spatial statistics-machine learning-mechanism analysis,” addressing the constraints of macro-level studies that frequently fail to provide specific policy recommendations. RECE is spatially and temporally depicted in ArcGIS. At the same time, a random forest model is used to assess the relative significance of BEFs, thereby suggesting micro-level residential carbon-reduction planning solutions. Key findings: (1) From 2021 to 2023, residential electricity carbon emission intensity (RECEI) and average annual household electricity carbon emissions (AHECE) demonstrated a trend toward concentrated, contiguous residential units with high building density, indicative of ongoing emissions reduction pressures in residential sectors; (2) High-emission units display notable spatial clustering and diffusion, primarily concentrated in the southern region of the study area, whereas low-carbon units are mainly located in peripheral zones; (3) In terms of driving factors, transportation compactness (TC) is the predominant influence, while adjusted land area (SA) and building density (BD) exert a moderating effect on residential electricity carbon emissions, suggesting that traditional high-intensity development metrics still possess potential for carbon mitigation. This paper proposes targeted low-carbon development strategies for residential electricity consumption based on these findings: optimizing integrated transportation networks, judiciously regulating building density and floor area ratio, systematically planning public service facilities, and augmenting green infrastructure. These optimization pathways are based on the inherent mechanisms linking transportation connectivity, spatial development intensity, and household power-related carbon emissions. They offer a pragmatic foundation for the design and development of low-carbon communities while concurrently facilitating regional carbon-reduction initiatives.

## 1 Introduction

Since the Industrial Revolution in 1850, the extensive use of fossil fuels and the persistent release of greenhouse gases have exacerbated global warming, resulting in various environmental issues such as extreme heat, heavy rainfall, droughts, and ecological degradation, which significantly threaten the sustainable development of human society (1, 2). The intergovernmental panel on climate change (IPCC) said in its Sixth Assessment Report (AR6) that the global average surface temperature increased by approximately 1.1 °C from 2011 to 2020 relative to pre-industrial levels (1850–1900) (3). Should this warming trend persist, the incidence of extreme weather events and the likelihood of ecological destabilization would escalate (4). In this context, regulating greenhouse gas emissions—primarily carbon dioxide (CO_2_)—and facilitating the low-carbon transition have emerged as essential concerns for the global community in tackling climate change.

In recent years, numerous nations and regions worldwide have established carbon peak and carbon neutrality objectives to optimize energy systems and advance green, low-carbon growth China, as one of the world's largest developing nations and a major carbon emitter, officially announced its “dual carbon” strategic objectives in 2020: to reach peak carbon emissions by 2030 and achieve carbon neutrality by 2060 (5, 6). With the ongoing increase in China's urbanization rate, cities have emerged as the principal geographical drivers of energy consumption and carbon emissions, particularly evidenced by substantial growth in residential power consumption (7).

Residential communities, as fundamental components of urban environments, generate carbon emissions at various stages of residents' daily activities, including building operations, household energy use, and daily commuting, thereby constituting the second-largest source of carbon emissions after the industrial sector (8). The composition of carbon emissions in residential communities is complex, encompassing emissions from household energy use, resident transportation, and building operations (9). Carbon emissions from building operations generally arise from the manufacturing of building materials, construction activities, and maintenance of buildings (10). The emissions span several steps, making accurate measurement challenging; moreover, certain emissions have previously been included in the industrial production and transportation sectors, complicating their differentiation (11). Carbon emissions from residential travel primarily come from private vehicles and public transit. These emissions primarily arise from the utilization of non-renewable energy sources, such as petroleum. Moreover, inhabitants' modes of transportation exhibit considerable variability and are profoundly subjective, complicating quantification (7). Conversely, carbon emissions from residential energy use predominantly stem from routine activities such as lighting, heating, and cooling, which are characterized by distinct parameters and robust measurability (12). Specifically, carbon emissions from domestic electricity use—the most immediate source of carbon emissions during the operational period of residential zones—have emerged as a central concern in urban low-carbon research (13). Current research demonstrates that elements of the built environment, building features, and resident behavior substantially affect home energy use and related carbon emissions (12). Consequently, clarifying the mechanisms by which built environment elements affect household energy-related carbon emissions is essential for formulating effective urban emission-reduction plans (14).

The built environment serves as a crucial intermediary between people's behavior and energy use, significantly influencing residential carbon emissions by shaping lifestyles, travel patterns, and microclimatic conditions (15). In recent years, investigations into the constructed environment and residential carbon emissions have advanced, largely developing into three principal study trajectories (16). Initially, research examines how residential building design, configuration, and operations influence energy consumption and carbon emissions; findings suggest that the arrangement of residential clusters significantly affects these metrics (17). Secondly, the research examines the issue through the lens of urban planning and management policies, scrutinizing the mechanisms by which elements such as land use, architectural design, and greenery affect carbon emissions (18); thirdly, it employs innovative technologies and models to monitor and assess carbon emissions, aiming to identify optimization pathways (19).

Existing studies employ a variety of methodologies, each with distinct limitations. Certain scholars utilize methodologies like the Grossman decomposition model to examine the effects of alterations in urban form on carbon emissions (18); others apply random forest models for importance analyses of built environment variables to discern critical influencing factors; additionally, some investigate the matter through the triad of density, function, and form to assess the influence of the built environment on carbon emissions stemming from urban construction land (20). Moreover, certain researchers have undertaken systematic investigations to elucidate the mechanisms by which the built environment affects residential electricity consumption, suggesting optimization strategies including density, housing unit design, and spatial configuration (21, 22). In contrast, others have examined the low-carbon potential of older residential districts and have recommended energy conservation and carbon-reduction measures through methodologies such as literature reviews, field surveys, and questionnaire interviews (23).

Despite existing research demonstrating the influence of the built environment on residential energy consumption and carbon emissions from various perspectives, and producing significant insights into the mechanisms of residential electricity usage and low-carbon renovation strategies for aging residential communities, two primary deficiencies persist. Initially, concerning data accuracy, carbon emission accounting and mechanism analyses at the home level predominantly depend on conventional data sources, like questionnaires, sample statistics, or scenario simulations. This complicates the attainment of continuous, objective, and precise characterization of energy use at the micro-spatial scale; data availability and timeliness are inadequate, and the outcomes are susceptible to subjective bias. Secondly, regarding study methodologies, the mechanisms by which the built environment affects residential carbon emissions often exhibit considerable non-linearity and spatial variability. Conventional statistical models struggle to balance predictive accuracy, explanatory mechanisms, and spatial effects in the presence of high-dimensional variables, substantial multicollinearity, and spatial dependencies, thereby limiting the application of research findings to planning, governance, and targeted policy initiatives. Consequently, in light of the “dual carbon” objectives, there is an imperative to implement micro-level energy consumption data with enhanced precision and extensive coverage, as well as to develop an analytical framework that can discern spatial heterogeneity, capture non-linear relationships, and provide interpretability, to facilitate the low-carbon transformation of residential communities and optimize policy.

The use of advanced metering infrastructure (AMI) offers novel opportunities to address these difficulties. AMI functions as the fundamental infrastructure of the smart grid system, encompassing a contemporary electricity metering and data collection framework that includes smart meters, communication networks, and backend data management systems (MDMS), which gather data on electricity consumption, usage periods, and variations in energy consumption (24). AMI data offers benefits such as objectivity, granularity, and continuity. Integrating AMI data into urban household carbon emissions research can offer empirical evidence for carbon emissions accounting, mitigate the impact of subjective factors on data quantification, and concurrently conserve substantial people and material resources. Nonetheless, the utilization of AMI data in current research has predominantly focused on the electricity sector, exhibiting a deficiency in multidisciplinary cross-application within carbon emissions research; this domain remains significantly underexplored in urban carbon emissions studies (25).

This study utilizes micro-scale residential electricity consumption data from State Grid's AMI to develop a methodological framework that combines spatial statistical analysis, machine learning modeling, and impact mechanism analysis, thereby addressing the deficiencies in current research. This paradigm addresses the limits of field-measured samples without substantially raising computing expenses, while concurrently rectifying the assumption biases present in conventional econometric models and alleviating the impact of outliers and noise interference. Moreover, it improves the interpretability of results via significance assessment. This framework presents micro-scale options for low-carbon development and carbon reduction in urban residential communities, focusing on domestic electricity use, and offers novel insights into research on carbon emissions in these areas.

The precise steps of this study are outlined as follows: initially, we computed the carbon emissions data associated with electricity for 146 residential communities (5,256 panel samples) in Wuqing District, Tianjin, from 2021 to 2023; Secondly, utilizing ArcGIS, we illustrated the spatial distribution characteristics of electricity carbon emissions data; Thirdly, we develop an evaluation index system for the built environment and a random forest model to systematically analyze the influence of built environment factors on residential electricity carbon emissions, conducting multiple comparative validations with carbon emission data from 2021 to 2023; Fourth, we develop pragmatic policy proposals for low-carbon retrofitting of home electricity carbon emissions in densely populated urban regions. This paper will concentrate on three fundamental questions. What is the regional distribution and annual fluctuation of carbon emissions from home power usage based on AMI data? What are the methods by which built environment elements influence household electricity carbon emissions? How might the research findings be converted into actionable planning and governance initiatives for densely populated metropolitan areas?

## 2 Materials and methods

### 2.1 Study area

**Figure 1:**
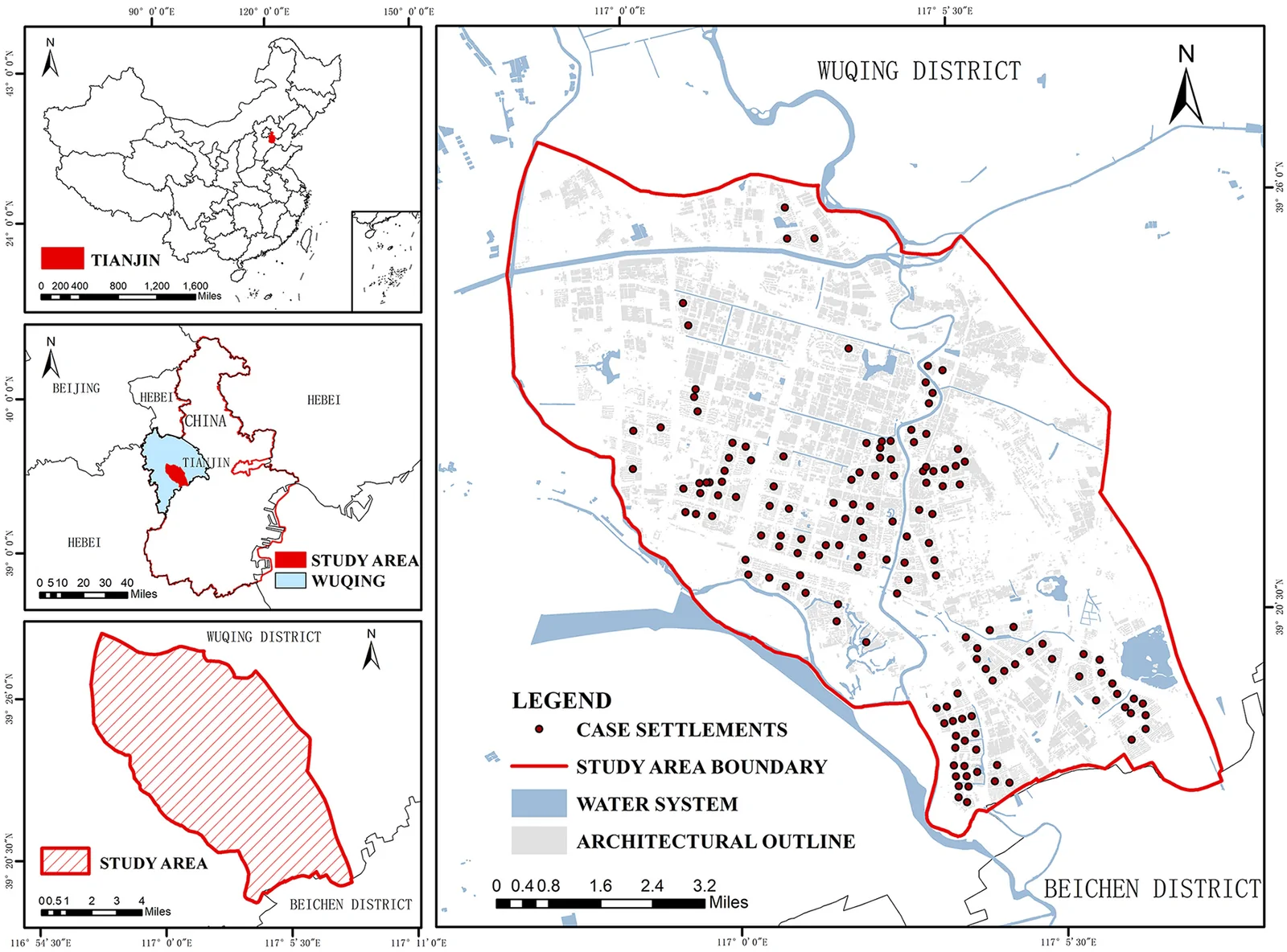
Location map of Wuqing District.

Tianjin (38°34′-40°15′ N, 116°43′-18°04′ E) is located in the northeastern region of the North China Plain, along the western coastline of Bohai Bay. As a prominent industrial center in northern China, its economy was traditionally supported by sectors such as steel and chemicals. In light of recent advances in the “dual carbon” approach, the city is expediting the transformation of its energy framework and the reconfiguration of low-carbon environments, establishing itself as a quintessential case study for research on carbon emissions in urban residential communities. Tianjin Municipality consists of 16 districts covering an area of 11,917 square kilometers. At the end of 2024, the permanent resident population was 13.64 million, comprising 11.73 million urban residents and 1.91 million rural residents, for an urbanization rate of 86.01%. The research area is situated in the central urban region of Wuqing District, in the northwestern section of Tianjin (Figure 1). The 146 residential communities identified in Wuqing District are predominantly situated in the central-western and southern sections, with a lesser concentration in the northern and eastern portions. The study area is a suburban region linking Tianjin to the capital, Beijing. The area is mostly comprised of recently constructed residential zones, where carbon emissions from residential and transportation sources have risen significantly, adversely affecting public health. Consequently, designating Wuqing District as the study region is a representative selection.

### 2.2 Research data

This study utilizes monthly residential electricity consumption data for Wuqing District, Tianjin, from 2021 to 2023, alongside administrative boundary data at provincial, municipal, and county levels; land use data; building outline data; road network data; Tianditu satellite imagery; park and green space data; and points of interest (POI) data. The smart meter electricity consumption data for residential communities were obtained from State Grid Tianjin Electric Power Company, the land-use data were supplied by the Tianjin Municipal Bureau of Planning and Natural Resources, and the remaining data were obtained from publicly accessible geospatial datasets on online platforms (Table 1).

**Table 1:** Data sources and description.

Name	Description	Sources
Electricity consumption data	Electricity consumption data of residential communities	State Grid Tianjin Electric Power Company (2023)
Land use data	Tianjin territorial spatial master plan data	Tianjin Municipal Bureau of Planning and Natural Resources
Building data	Building footprint information (building area, height, type, etc.)	Housing and urban-rural development commission
Road data	Vector data of the urban road traffic system	OpenStreetMap (OSM)
Population data	Number of households in residential communities	Anjuke website
Remote sensing data	Tree canopy coverage data	https://www.gscloud.cn/
POI data	Location information of public transportation stops, public service facilities, educational service facilities, and commercial facilities	Baidu Maps

This research concentrates on the core urban region of Wuqing District in Tianjin, analyzing 225 residential neighborhoods within that locale. To guarantee that the sample adhered to the research criteria of being exclusively residential, possessing continuous time-series data, and exemplifying a typical and manageable constructed environment, this study formulated explicit and replicable three-tiered sample selection guidelines. Communities were evaluated systematically according to three criteria: the thoroughness of monitoring data, the integrity of land use characteristics, and the typicality and regional relevance of the constructed environment, with strict exclusion of those failing to satisfy the study's definitions and data prerequisites. In the end, 146 legitimate study samples were acquired. Initially, assessing the comprehensiveness of time-series electricity usage data. Established residential neighborhoods with uninterrupted AMI smart electricity monitoring data for the entirety of 2021–2023—lacking significant data voids or prolonged transmission disruptions—were selected; areas under construction, in the initial phases of new occupancy, or with insufficient monitoring equipment leading to fragmented annual time-series data were omitted. Secondly, evaluating the integrity of land-use characteristics. Communities that combine business and residential uses, featuring a significant amount of commercial space, a mixture of commercial and residential activities, or where electrical loads could not be distinctly divided, were omitted. This measure was implemented to prevent disruption from commercial electricity usage in the assessment of carbon emissions from residential electricity consumption and to guarantee that the sampled electricity data precisely represented home energy consumption trends. Third, evaluating the typicality of the constructed environment and the regional representativeness. To guarantee that the research sample authentically represents the general characteristics of the built environment in residential communities within the study area, this research integrated remote sensing imagery, urban functional zoning, and data on residential construction periods to systematically eliminate atypical residential communities distinguished by unique built environments, significant disparities in housing types and amenities, or specialized district functions. This phase seeks to ensure that the attributes of the constructed environment for all legitimate samples are broadly relevant, facilitating regional empirical examination, while preventing potential distortion of recognized trends by atypical samples. Additionally, to enhance validation of sample representativeness, this research analyzed the spatial distribution across the entire study region and the diversity of residential categories among the 146 valid samples, thereby confirming whether the samples included all standard residential area types within the study area. The valid samples exhibit a uniform representation of mature and older residential neighborhoods within the central urban region of Wuqing District; premium commercial residential zones in the core area; suburban transitional housing areas; and residential locales in new towns established for over 3 years. The research region thoroughly encompasses residential communities that exemplify various phases of urban evolution, construction periods, availability of amenities, and spatial configurations. Consequently, they can comprehensively and impartially represent the overarching characteristics of the built environment and the spatial differentiation patterns of residential communities in Wuqing District, thereby offering a dependable data foundation for future analyses concerning the correlation between built environment elements and electricity usage and carbon emissions.

This study employed the ArcGIS platform to spatially integrate calculations of residential electricity carbon emissions with residential area attributes and current land-use data, thereby creating a comprehensive spatial database to examine the distribution patterns of residential electricity carbon emissions and their underlying mechanisms.

### 2.3 Methods

#### 2.3.1 Measurement of residential electricity carbon emissions (RECE)

The research examines carbon emissions arising from household electricity usage. RECE is computed using Equation 1, utilizing carbon emission factors and measuring electricity consumption per residential neighborhood as the unit.


$$\begin{array}{l} E=AD\times EF \end{array}$$ (1)

where *E* is carbon emissions (kg), *AD* is electricity consumption, and *EF* is the carbon emissions coefficient. Among them, *AD* is the annual electricity consumption of residential land provided by the Tianjin power supply company.

EF denotes the Guidelines for National Greenhouse Gas Inventories released by the Intergovernmental Panel on Climate Change (IPCC) in 2006, the CO_2_ emission factors for electricity provided by the Ministry of Ecology and Environment, and the Draft for Public Comment on the Standards for Carbon Footprint Calculation and Carbon Labeling Assessment of Roof Greening Modules. These criteria utilize the national average carbon dioxide emission factor for electricity and omit electricity derived from non-fossil energy sources acquired via market exchanges. It is important to highlight that Tianjin is integrated into the North China Regional Power Grid and is characterized by a power generation composition that is mostly reliant on combined heat and power (CHP) and coal-fired energy, which markedly differs from the national average. Utilizing the national average factor would consequently result in a degree of systematic distortion concerning the true carbon emission profile of electricity in Tianjin. Nevertheless, considering the research aims and empirical rationale of this investigation, the uniform application of the national average emission factor is both scientifically valid and justifiable. The primary aim of this research is to elucidate the comparative influence mechanisms and contribution degrees of variations in the built environment among different residential communities on carbon emissions resulting from residential electricity use, emphasizing cross-sectional comparisons and pattern recognition among samples, rather than the exact quantification of absolute regional carbon emissions. In light of this research aim, the foremost criterion for choosing the emission factor is to guarantee the uniformity of the 146 residential community samples within a single accounting framework, thus reducing the impact of regional variations in electricity generation composition on the outcomes of spatial analyses. The national average electricity emission factor serves as a uniform measurement standard for all samples, effectively eliminating systematic biases arising from regional variations in electricity composition and thereby elucidating the fundamental connection between built environment elements and carbon emissions from electricity use. Moreover, since this element was collaboratively published by the Ministry of Ecology and Environment and the National Bureau of Statistics, it carries credibility and replicability, enabling cross-sectional analyses and verification of findings in future research. In conclusion, employing the national average electricity CO_2_ emission factor in this research strikes a judicious equilibrium among research aims, sample uniformity, and data credibility. Subsequent investigations might amalgamate regional power grid emission coefficients and electricity composition data to improve the accuracy of carbon emission assessments for residential zones.

The statistics and sources for the national average electricity CO_2_ emission factor (excluding non-fossil energy power generated via market-based transactions) utilized in this paper are presented below (Table 2):

**Table 2:** Electricity carbon emission coefficient.

Year	Region	Average electricity emission factor (kgCO_2_/kWh)	Source
2021	China	0.5942	Announcement of the Ministry of Ecology and Environment and the National Bureau of Statistics on the Release of Carbon Dioxide Emission Factors for Electricity in 2021.
2022	China	0.5856	Announcement of the Ministry of Ecology and Environment and the National Bureau of Statistics on the Release of Carbon Dioxide Emission Factors for Electricity in 2022.
2023	China	0.5703	Carbon footprint accounting and carbon labeling evaluation standard for green roof modules (Draft for comments).

Residential electricity carbon emission intensity (RECEI) is the ratio of residential electricity carbon emissions (RECE) to the residential area. Annual household electricity carbon emissions (AHECE) is the quotient of RECE and the total number of households in the residential area. The methodologies for measuring RECEI and AHECE are delineated as follows:


$$\begin{array}{l} I=E/S \end{array}$$ (2)

where *I* is carbon emissions intensity (kg/m^2^), *E* is carbon emissions (kg), and *S* is the area of residential land (m^2^).


$$\begin{array}{l} A=E/H \end{array}$$ (3)

where *A* is average household carbon emissions (kg/h), *E* is carbon emissions (kg), and *H* is the number of households in the RB (h).

#### 2.3.2 Measurement of built environmental factors (BEF)

Prior research indicates that the urban built environment significantly influences carbon emissions from home electricity usage. Principal contributing elements include land-use intensity, architectural design, transportation conditions, ecological and environmental attributes, and accessibility to public service facilities. This research identifies and evaluates influencing factors across three spatial scales—the building, intra-neighborhood, and surrounding environment scales—to verify that the selected indicators effectively capture the pathways of influence across these scales. The architectural form predominantly affects indoor thermal conditions and energy consumption; the internal spatial configuration and land use attributes of a neighborhood modify local microclimates, development density, and ventilation conditions, consequently impacting residential electricity usage; and the surrounding built environment indirectly influences carbon emissions related to residential electricity by altering residents' daily activities and energy consumption behaviors through the accessibility of public services and transportation infrastructure. The three scales described are exclusively used to clarify the origins of indicators and their influence pathways; they do not constitute distinct classifications of indicators at each level.

This article classifies the evaluation indicator system for the built environment of residential neighborhoods in Wuqing District, Tianjin, into four principal dimensions: land use intensity, transportation configuration, ecological environment, and public services. Fourteen quantifiable aspects of the built environment are selected as evaluation indicators across these four dimensions, including floor area ratio, building density, building form factor, transportation compactness, tree coverage, and land use mix (see Table 3 for specific indicators). The previously mentioned four-dimensional indicator system systematically characterizes variations in the built environment of residential communities, establishing a clear and consistent foundation for subsequent model-based identification of influential factors and analysis of their mechanisms of action.

**Table 3:** Evaluation index system of the built environment in Wuqing District.

Type	BEF	Definition	Formula	Description
Land Use Intensity	Floor area ratio (FAR)	FAR is the floor area ratio of the residential area	$FAR=\frac{\overset{n}{\underset{i=1}{\sum }}M_{i}}{S_{j}}$	*n* is the number of individual buildings within the residential area; *M*_*i*_ is the total floor area of the *i*-th building within the residential area; *S*_*j*_ refers to the land area of the *j*-th residential community.
	Land area (SA)	SA represents the total land area of the residential community	*SA* = *S*_*j*_	*S*_*j*_ refers to the land area of the *j*-th residential community, and each *S*_*j*_ is separately extracted from GIS spatial vector data.
	Building density (BD)	BD is the building density within the residential area	$BD=\frac{\overset{n}{\underset{i=1}{\sum }}S_{i}}{S_{j}}$	*n* is the number of individual buildings within the residential area; *S*_*i*_ is the base area of the *i*-th building within the residential area; *S*_*j*_ refers to the land area of the *j*-th residential community.
	Building configuration (BCF)	BCF is the building arrangement type within the residential area	*BCF* = *X*_*i*_, *i* ∈ {1, 2, 3, 4, 5}	*X*_*i*_ is the *i*-th type of building configuration, where 1 = detached, 2 = scattered, 3 = enclosed, 4 = row-and-column, and 5 = mixed arrangement.
	Building form factor (BSC)	BSC is the building shape coefficient	$BSC=\frac{\overset{n}{\underset{i=1}{\sum }}\left[2m_{i}h_{i}\left(b_{i}+l_{i}\right)+S_{i}\right]}{\overset{n}{\underset{i=1}{\sum }}m_{i}h_{i}S_{i}}$	*n* is the number of buildings on the plot; *m*_*i*_ is the number of stories in the *i*-th building; *h*_*i*_ is the story height of the *i*-th building; *b*_*i*_ is the base width of the *i*-th building; *l*_*i*_ is the base length of the *i*-th building; *S*_*i*_ is the base area of the *i*-th building.
	Urban road network density (URND)	URND is the road network density within a 1 km radius of the residential area	$URND=\frac{\overset{n}{\underset{i=1}{\sum }}L_{i}}{S_{r}}$	*n* is the number of roads within the radius; *L*_*i*_ is the length of the *i*-th road within the radius; *S*_*r*_ is the area of the 1 km radius around the residential area.
Transportation Layout	Transportation compactness (TC)	TC is the residential area's transportation compactness	$TC=\frac{S_{l}}{H}$	*S*_*l*_ is the area of urban roads within a 1 km radius centered on the geometric center of the residential area; *H* is the number of households in the residential area.
	Public transportation stop density (PTSD)	PTSD is the public transportation stop density of the residential area	$PTSD=\frac{R}{S_{r}}$	*R* is the number of public transportation stops within a 1 km radius of the residential area; *S*_*r*_ is the area within a 1 km radius of the residential area.
Ecological environment	Green space ratio (GSR)	GSR is the green space ratio of the residential area	$GSR=\frac{\overset{n}{\underset{i=1}{\sum }}SG_{i}}{S_{j}}$	*n* is the total number of green spaces within the residential area; *SG*_*i*_ is the area of the *i*-th green space within the residential area; *S*_*j*_ refers to the land area of the *j*-th residential community.
	Tree coverage rate (ACR)	ACR is the tree coverage ratio of the residential area	$ACR=\frac{ST_{i}}{S_{j}}$	*ST*_*i*_ is the area of the ground projection of the crown of the *i*-th tree in the residential area; *S*_*j*_ refers to the land area of the *j*-th residential community.
	Proportion of green space (GAP)	GAP is the proportion of green space area in the residential area	$GAP=\frac{A_{EG}}{A_{E}}$	*A*_*EG*_ is the green space area within a 1 km radius centered on the geometric center of the residential area; *A*_*E*_ is the total land area of the 1 km radius.
Public services	Diversity of public service facilities (DPSF)	DPSF is the diversity of public service facilities in the residential area	$DPSF=\overset{n}{\underset{i=1}{\sum }}\frac{K_{i}}{S_{r}}$	*n* is the total number of public service facility categories within the area; *K*_*i*_ is the number of public service facilities of the *i*-th category within the area; *S*_*r*_ is the 1 km area of the residential area.
	Accessibility of educational facilities (AEF)	AEF is the accessibility of educational facilities in the residential area	*AEF* = min(*LE*_*i*_)	*LE*_*i*_ the length of the travel route from the *i*-th residential area to the corresponding elementary school in the school district.
	Land use mix (LUMD)	LUMD is the land use mix in the residential area	$LUMD=-\overset{u}{\underset{i=1}{\sum }}\left(\frac{W_{i}}{U}\right)\ln\left(\frac{W_{i}}{U}\right)$	*u* is the total number of POI categories within the area; *W*_*i*_ is the number of POIs of the *i*-th category within the area; *U* is the total number of POIs within a 1 km radius of the residential area.

#### 2.3.3 Gray correlation analysis method

Gray Correlation Analysis (GCA) was introduced by Deng in 1982 and is mostly employed to examine the interrelations among components in a system characterized by insufficient information and restricted sample size (26). Gray Correlation Analysis Method discerns possible correlation traits among variables by evaluating the geometric resemblance of trends across various variable sequences. It places no stringent demands on data distribution and is appropriate for urban environmental studies that encompass uncertainty and intricate interconnections (27, 28). This research utilizes it for an initial investigation into the extent of correlation between building environmental factors and residential electricity carbon emissions.

In this study, carbon emissions associated with residential electricity carbon emissions (RECE) are designated as the dependent variable (*y*), while many aspects of the building environmental factors (BEF) are classified as independent variables (*x*). This research identifies the key environmental factors driving regional disparities in residential electricity carbon emissions by assessing the gray association between various built-environment indicators and carbon emissions. In accordance with the methodology suggested by Wu et al. (29), the computational formulas are delineated in Equations 4–7.


$$\begin{array}{l} C\left(t\right)=\frac{1}{m\times n}\overset{m}{\underset{i=1}{\sum }}\overset{n}{\underset{j=1}{\sum }}\varepsilon_{ij}\left(t\right) \end{array}$$ (4)

In the equation, *C*(*t*) denotes the correlation degree between building environmental factors and residential electricity carbon emissions; *m* signifies the quantity of building environmental factors; *n* indicates the number of residential area samples; and ε_*ij*_(*t*) represents the gray correlation coefficient. The research utilized data standardization techniques to mitigate dimensional interference and enhance computational precision (30). The formula for determining the gray correlation coefficient is presented in Equation 5:


$$\begin{array}{l} \varepsilon_{ij}\left(t\right)\\ =\frac{\min_{i}\min_{j}|Z_{i}^{X}\left(t\right)-Z_{j}^{Y}\left(t\right)|+\rho \cdot \max_{i}\max_{j}|Z_{i}^{X}\left(t\right)-Z_{j}^{Y}\left(t\right)|}{|Z_{i}^{X}\left(t\right)-Z_{j}^{Y}\left(t\right)|+\rho \cdot \max_{i}\max_{j}|Z_{i}^{X}\left(t\right)-Z_{j}^{Y}\left(t\right)|} \end{array}$$ (5)

In the equation, $Z_{i}^{X}\left(t\right)$ denotes the normalized value of the *i*th building environmental factors; $Z_{j}^{Y}\left(t\right)$ signifies the normalized value of carbon emissions for the *j*th residential area sample; ρ is the discrimination coefficient, established at ρ = 0.5 in this study. Utilizing the approach suggested by Olu-Ajayi et al. (30), the range-normalization technique is employed to derive the normalized sequences $Z_{i}^{X}\left(t\right)$ and $Z_{j}^{Y}\left(t\right)$. The formula for calculation is presented in Equation 6:


$$\begin{array}{l} Z_{ij}=\frac{X_{ij}-\min_{i}X_{ij}}{\max_{i}X_{ij}-\min_{i}X_{ij}} \end{array}$$ (6)

By averaging the gray correlation coefficients across all residential area samples, we obtain the overall gray correlation (29) between each building's environmental factors and residential electricity carbon emissions. The formula for calculation is presented in Equation 7:


$$\begin{array}{l} \gamma_{ij}=\frac{1}{n}\overset{n}{\underset{j=1}{\sum }}\varepsilon_{ij}\left(t\right) \end{array}$$ (7)

In the formula, *n* denotes the total number of samples; γ_*ij*_ denotes the gray correlation coefficient linking the *i*th building environmental factors to residential electricity carbon emissions. The greater the value, the more robust the association between the two. Per conventional classification criteria: 0.8 < γ < 1 signifies a very robust correlation; 0.6 < γ < 0.8 denotes a strong correlation; 0.3 < γ < 0.6 reflects a moderate correlation; and 0 < γ < 0.3 represents a weak correlation (31).

Gray correlation analysis can initially indicate the extent of alignment in the changing trends of different building environmental factors and carbon emissions; nonetheless, it fails to directly measure the proportional impact of each variable on carbon emissions and cannot precisely depict the non-linear response relationships between the variables and carbon emissions. Consequently, this research expands on gray correlation analysis by using a random forest model to assess the relative impacts of various building environmental factors on residential electricity carbon emissions, leveraging an ensemble learning algorithm, and examining their non-linear influence characteristics.

#### 2.3.4 Random forest model

This research builds on the initial validation from the preceding section's gray correlation analysis, which revealed a non-linear association between various building environmental factors and residential electricity carbon emissions, by incorporating a random forest model to assess the significance of each factor. The rationale for employing gray correlation analysis alongside the Random Forest model is that the former merely identifies “whether” factors correlate with carbon emissions and “to what degree” they interact, yet fails to elucidate the independent contribution of each variable to variations in carbon emissions when multiple factors are present—addressing this inquiry holds greater practical significance for pinpointing essential planning and regulatory components (32, 33). Random Forest is an ensemble learning technique derived from decision trees, first introduced by Breiman in 2001 (32). The fundamental tenet entails employing a bootstrap sampling technique to randomly extract many subsamples with replacement from the initial training dataset (32). For every subsample, an individual decision tree is generated, and the final predictions of all decision trees are then combined (averaging for regression tasks and using the mode for classification tasks) to yield the model's ultimate forecast. This collective approach makes Random Forest markedly more effective than an individual decision tree in terms of predictive precision and model robustness. Moreover, at the node division phase of each tree, Random Forests do not identify the most effective splitting variable from the entire collection of predictors; rather, they select the optimal splitting variable from a randomly chosen subset of predictors. This process diminishes the correlation among trees and significantly improves the model's generalization capacity (33). In practical scenarios, Random Forests exhibit significant robustness to high-dimensional datasets, non-linear associations, and variable interactions, necessitating minimal data preprocessing; consequently, they are extensively utilized in areas like ecological systems, urban climatology, and the detection of carbon emission determinants (34).

The development of a Random Forest model can be implemented using several prevalent software tools; MATLAB, Python, and R are frequently used. Given the data attributes and research specifications of this study, MATLAB was ultimately chosen to construct the Random Forest model. In the model generation process, RECE served as the dependent variable. In contrast, BEF at multiple spatial scales served as the independent variable, using *K*-fold bootstrap sampling to analyze the sample set.

To maintain the consistency of analytical outcomes throughout many years and the reliability of model efficacy, the Random Forest models for 2021–2023 were established using standardized parameters. The parameters were established by observing the pattern of out-of-bag error as the number of decision trees grew (35). Experiments indicated that after the tree count reached 600, the out-of-bag error curve stabilized, and every subsequent increase in trees yielded an accuracy enhancement of under 0.5%; thus, the decision tree quantity was established at 600. The minimum leaf node size (MinLeafSize) was established at 5 to constrain the intricacy of each decision tree and mitigate the likelihood of overfitting; Given that the quantity of built environment indicators preserved post-feature selection was moderate, all predictors were incorporated in the partitioning process (NumPredictorsToSample = all) to consider possible interactions among variables (36); the model utilized the standard bootstrap sampling method, wherein the training set for each tree was produced through with-replacement random sampling, maintaining a sample size equivalent to that of the original training set. The out-of-bag prediction capability was activated to assess the model's generalization robustness. The significance of variables was determined by the mean increase in out-of-bag replacement error, with importance scores normalized to percentages for cross-year comparisons (35); a random seed of 2024 was set to ensure reproducibility of Bootstrap sampling and model training outcomes. Furthermore, before modeling, all continuous predictor variables underwent z-score standardization to remove the influence of unit discrepancies on variable importance assessment (37); the dependent variable was log1p-transformed to alleviate the effects of its right-skewed distribution on model fitting (38). Data partitioning used a stratified random sampling technique, dividing the dataset into training and testing subsets at a predetermined ratio of 75% to 25%. The distribution of samples for both the training and testing datasets each year was generated using the same random seed (2024) to maintain consistency in partitioning outcomes across years. The previously mentioned parameter configurations and preprocessing methods were consistently utilized throughout the independent models for 2021, 2022, and 2023 to guarantee the dependability and comparability of the cross-year analytical outcomes. This research developed distinct random forest models for 2021, 2022, and 2023, rather than employing a panel data model, primarily for methodological reasons. The principal benefit of panel data models is their ability to estimate the average influence of variables over time while accounting for individual differences (39); nonetheless, this research primarily emphasizes the explanatory capacity and comparative contributions of different built environment elements to residential electricity carbon emissions at a cross-sectional level, along with an annual assessment of the importance hierarchy of these elements and their trends over the years. As standard “slow variables,” they demonstrate significant multicollinearity with specific fixed effects, complicating the accurate estimation of their coefficients in panel models. Conversely, yearly cross-sectional modeling can directly leverage the geographical variability of the constructed environment over time to accurately assess the significance of each element. Consequently, by preserving uniformity in model parameter configurations, data processing methodologies, and random seeds across all 3 years, this research independently analyzes the data for each year to ensure comparability and reproducibility of the findings.

## 3 Results

### 3.1 Characteristics of RECE

#### 3.1.1 Quantitative characteristics

Carbon emissions were computed using the formula and AMI data, and the results are presented in Table 4. Between 2021 and 2023, the AECE (Annual Average Electricity Carbon Emissions) demonstrated notable phased evolution. The 3-year averages were 1,731,243.432 kgCO_2_, 2,121,559.523 kgCO_2_, and 2,075,605.881 kgCO_2_, respectively, illustrating a temporal pattern of “rapid short-term rise followed by a gradual reduction and stabilization. In 2022, AECE rose by 22.55% compared to 2021, signaling a significant increase in carbon emissions from domestic energy consumption in the region. In 2023, there was a minor year-on-year decline of 2.17%, reducing the overall increase relative to 2021 to 19.89%. This indicates that home electricity demand in the research area is approaching saturation, and carbon emissions from residential electricity consumption have transitioned from a period of significant increase to a stable phase. The spatial distribution of localities with carbon emissions below the yearly average across 3 years was 69.19%, 68.49%, and 67.81%, constantly above 68%. This indicates a distorted spatial distribution marked by the aggregation of low-emission values and the paucity of high-emission values in the area. This study chose 10 sets of high- and low-value samples for each year of AECE to elucidate the reasons underlying this fluctuation through comparative analysis. The findings indicate that total community land area (SA), transportation compactness (TC), and accessibility of educational facilities (AEF) are the principal indicators influencing the variation in total carbon emissions, demonstrating a significant inverse correlation: as SA increases, TC decreases, and AEF diminishes, resulting in higher total carbon emissions from residential electricity consumption. Standard extreme-value samples validate this trend: Poly Shanghe Yasheng has consistently exhibited elevated carbon emissions in the region for three consecutive years, surpassing the annual norm by 486% to 510%. An elevated surface area signifies that a community can support a greater resident population. The outstanding location and complete amenities (TC, AEF) increase livability, attracting a concentration of permanent inhabitants. As a result, the diversity of household equipment possessed and overall daily power usage increase simultaneously, ultimately elevating the community's total RECE. Chenfeng Jiayuan (2021) and the Agricultural Machinery Bureau Dormitory (2022–2023) served as low-value samples for the year, with carbon emissions accounting for only 1.1%−4.1% of the regional average. The very limited surface area of these settlements restricts the maximum population capacity. Inadequate external amenities diminish appeal, resulting in a limited number of permanent residents and a restricted baseline for overall household electricity usage. This automatically constrains the maximum limit of the community's total carbon emissions associated with power, establishing a consistent pattern of fluctuation in overall carbon emissions.

**Table 4:** The value of AECE, AECEI, and AHECE.

Year	Variables	Sample size	Mean	Standard deviation	Maximum	Minimum
2021	RECE	146	1,731,243.432 (AECE)	1,782,402.179	10,201,024.17	18,826.6328
	RECEI	146	109.5440425 (AECEI)	704.0347478	8,091.901919	0.709951775
	AHECE	146	1,124.711088	479.4416805	2,465.069974	87.16033704
2022	RECE	146	2,121,559.523 (AECE)	2,097,108.92	12,943,085.21	86,967.456
	RECEI	146	158.7749756 (AECEI)	1,110.224194	12,957.88175	1.616991039
	AHECE	146	1,391.171508	524.4830064	3,046.510453	219.1117227
2023	RECE	146	2,075,605.881 (AECE)	2,004,636.183	12,171,352.3	68,162.256
	RECEI	146	171.4393853 (AECEI)	1,254.865428	14,765.12248	1.559932082
	AHECE	146	1,365.114688	508.355884	3,545.095617	289.2190581

AECEI (annual average carbon emission intensity per unit of electricity consumption) demonstrates a persistent upward trend, coupled with a decelerating growth rate. Between 2021 and 2023, the regional average increased annually from 109.54 kgCO_2_/m^2^ to 158.77 kgCO_2_/m^2^, then to 171.44 kgCO_2_/m^2^, representing a total increase of 56.50% over the three-year period. The yearly growth rate decreased from 44.94% to 7.98%, indicating a steady rationalization of energy consumption per unit area in communities, alongside ongoing improvements to the energy consumption structure. The carbon emission intensity in the investigated area shows significant spatial polarization. Between 2021 and 2022, 93.84% of towns had RECEI values below the annual average; by 2023, this percentage increased to 94.52%. The intensity levels of most communities approached equilibrium, with only a few ultra-high-intensity samples substantially elevating the regional average. This indicates that focused management of high-value communities can effectively reduce emissions. A comparison of 10 high-value and 10 low-value sample groups showed that, unlike the typical driving mechanisms identified with AECE, built environment indicators such as SA, TC, and AEF had minimal associations with carbon emission intensity. Carbon emissions per unit area are not predominantly influenced by macro-level factors such as community land area or local amenities. The disparities among extreme-value samples further validate the previously noted pattern: Huajun Jiayuan has achieved the highest ranking in the district for carbon emission intensity for three consecutive years, with values surpassing the yearly average by over 70 times. This neighborhood includes many residential types, such as high-rise, mid-rise, and townhouses, featuring considerable differences in unit design, living quality, and the arrangement of in-unit amenities. In contrast to conventional entry-level housing, villas and enhanced housing units offer more spacious interiors, superior appliance arrangements, and greater prevalence of comfort-oriented electrical systems, including climate control and fresh air systems. Inhabitants of these units exhibit more tailored electricity consumption patterns and use electricity more often. Due to the limitations of a given community land area, variations in unit configuration and housing types directly increase electricity demand per unit of floor area, thereby leading to a significantly high carbon emission intensity. Conversely, low-value communities such as Chenfeng Jiayuan and the Ministry of Water Resources and Electric Power South Compound exhibit homogeneous unit types with uncomplicated interior layouts. Electricity use is predominantly influenced by fundamental daily requirements, with a minimal fraction attributed to energy-intensive devices and comfort-related electricity demands. The electricity consumption patterns of residents are uniform, and energy usage is persistently low, leading to a minimal carbon emission intensity per unit area. This study indicates that discrepancies in AECEI are mostly influenced by variances in residential usage patterns and unit layout characteristics within communities. They exhibit limited relationships with macro-level built environment variables (SA, TC, AEF), such as exterior location and land area, leading to substantial divergence between the spatial evolution of carbon emission intensity and the underlying logic of total AECE.

The temporal progression of annual household electricity carbon emissions (AHECE) typically mirrors the trajectory of total carbon emissions, with an initial increase followed by a slight decrease as regional per-home power consumption progressively stabilizes. A notable convergence trend is apparent at the spatial distribution level. Between 2021 and 2023, the percentage of communities with AHECE below the yearly average rose from 44.52% to 46.58%, then to 49.32%. The gap in per-home electricity-related carbon emissions across residential regions is diminishing, and regional household energy consumption patterns are becoming increasingly uniform and equitable. An analysis of the traits of the highest and lowest 10 sample groups indicates that per-household carbon emissions are similarly not substantially influenced by macro-level built environment indicators such as SA, TC, and AEF; macro-spatial factors exhibit limited explanatory capacity concerning individual household electricity consumption patterns and energy usage levels. Throughout the study period, the Transportation Bureau Dormitory (2021) and Longwan Longyi Garden (2022–2023) represented high-value samples for per-household carbon emissions, whereas Chenfeng Home (2021) and Yonghua Garden (2022–2023) constituted low-value samples. An analysis of built environment parameters across 10 high-value and 10 low-value sample groups indicates that AHECE values show no significant correlation with macro-level built environment indicators such as SA, TC, and AEF, suggesting that community-scale built environment factors are insufficient to explain variations in per-household carbon emissions. This pattern aligns with the trait that the AECEI is not limited by macro-level built environment indicators. The three categories of carbon emission indicators exhibit distinct response patterns: macro-level built environment factors can substantially affect total electricity-related carbon emissions in residential zones, yet their explanatory capacity for carbon emission intensity per unit area and per-household carbon emissions is constrained. An extensive examination of quantification patterns across these three indicator categories demonstrates that a mechanism analysis of residential electricity-related carbon emissions, grounded in built environment indicators, is both theoretically and empirically viable, thereby establishing an empirical basis for the systematic identification of the carbon emission drivers outlined below.

#### 3.1.2 Spatial characteristics

**Figure 2:**
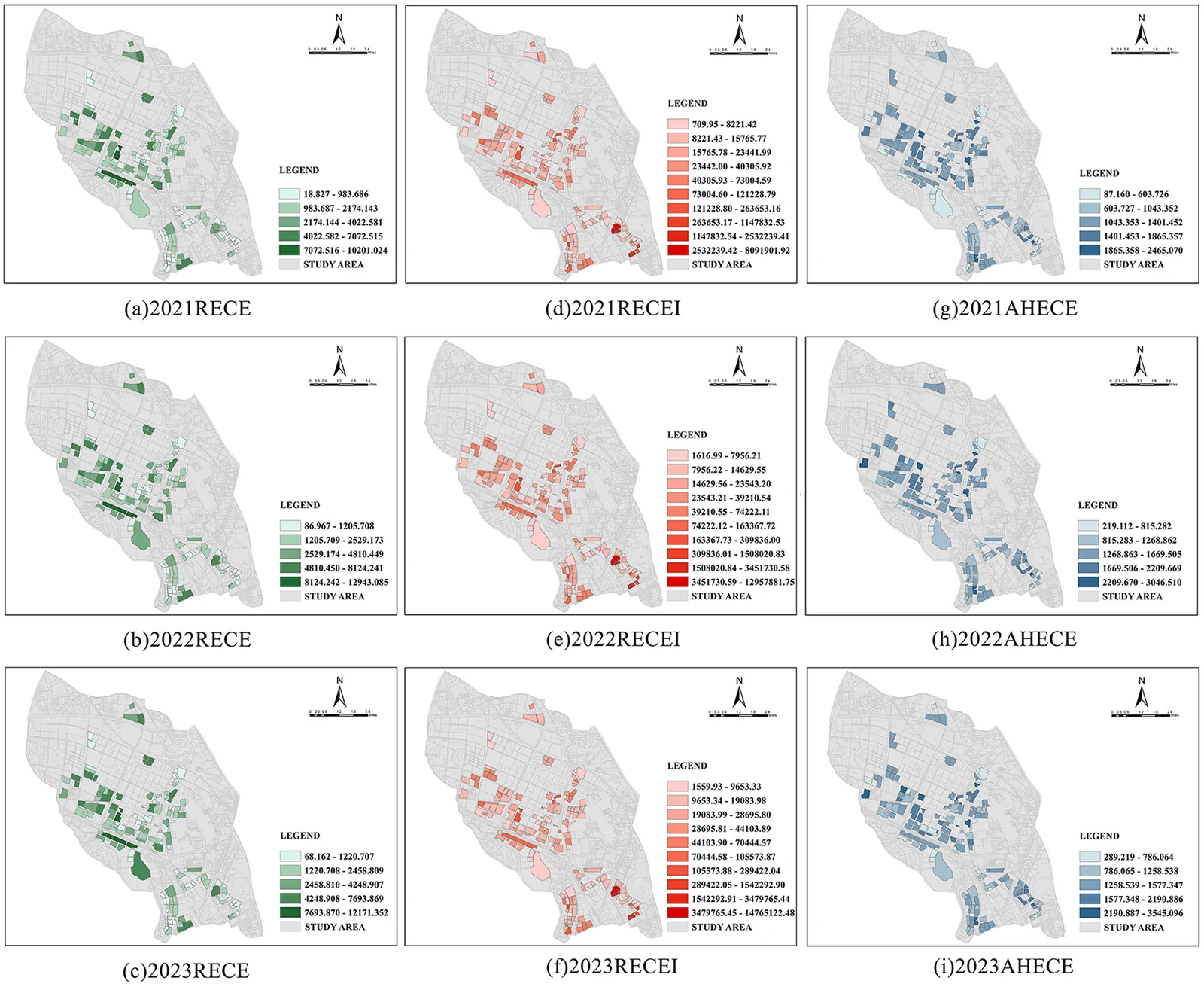
Spatial distribution maps of RECE, RECEI, and AHECE in the study area from 2021 to 2023. **(a)** Spatial distribution map of RECE in the study area in 2021. **(b)** Spatial distribution map of RECE in the study area in 2022. **(c)** Spatial distribution map of RECE in the study area in 2023. **(d)** Spatial distribution map of RECEI in the study area in 2021. **(e)** Spatial distribution map of RECEI in the study area in 2022. **(f)** Spatial distribution map of RECEI in the study area in 2023. **(g)** Spatial distribution map of AHECE in the study area in 2021. **(h)** Spatial distribution map of AHECE in the study area in 2022. **(i)** Spatial distribution map of AHECE in the study area in 2023.

ArcGIS software facilitated the visualization of carbon emissions calculations to examine the spatial distribution characteristics of three principal metrics—RECE, RECEI, and AHECE—across 146 residential communities in Wuqing District from 2021 to 2023 (Figure 2).

Between 2021 and 2023, the RECE spatial patterns of 146 residential communities in Wuqing District remained stable throughout the year, typically showing a distribution with elevated values in the north and lower values in the south. Communities with elevated RECE values were predominantly located in the center region of the research area, south of Cuiheng Road and adjacent to the intercity railway line, whereas those with diminished values were dispersed in linear formations along the North Canal, west of Nanhu Park, and west of Wenhui Road; In conjunction with the previously described quantitative findings regarding the built environment, communities situated along arterial highways demonstrate elevated SA values, enhanced AEF for amenity accessibility, and improved TC for locational conditions. Population concentration leads to an escalation in overall home energy use, which is the primary driver of the persistent increase in total carbon emissions in this region. The spatial distribution of RECEI remained predominantly stable over the three-year duration. High-value residential zones had no discernible clustering along thoroughfares, but low-value residential zones were dispersed intermittently throughout the territory. In conjunction with the previously described sample statistical results, externally built environment variables such as SA, TC, and AEF exhibited no significant link with carbon emission intensity, suggesting that the factors influencing regional variation in intensity differ from those affecting total carbon emissions. The interannual spatial fluctuations in AHECE are negligible, with a generally balanced, multi-core distribution. High-value residential zones are concentrated along the southern segment of Cuiheng Road and the central portion of the North Canal, developing in contiguous clusters, whilst low-value residential communities are predominantly dispersed. In conventional low-value regions like Guanlan Garden, carbon emissions have increased annually and are steadily nearing the median level. This spatial evolution aligns with the previously discussed convergence pattern of per-household carbon emissions: macro-level built environment conditions fail to limit individual household electricity consumption, and the standardization of residential energy use propels the persistent upward movement of regional low-value zones. An extensive investigation of regional differentiation patterns among the three indicator categories demonstrates that built environment elements have markedly distinct spatial impacts on residential carbon emissions.

**Figure 3:**
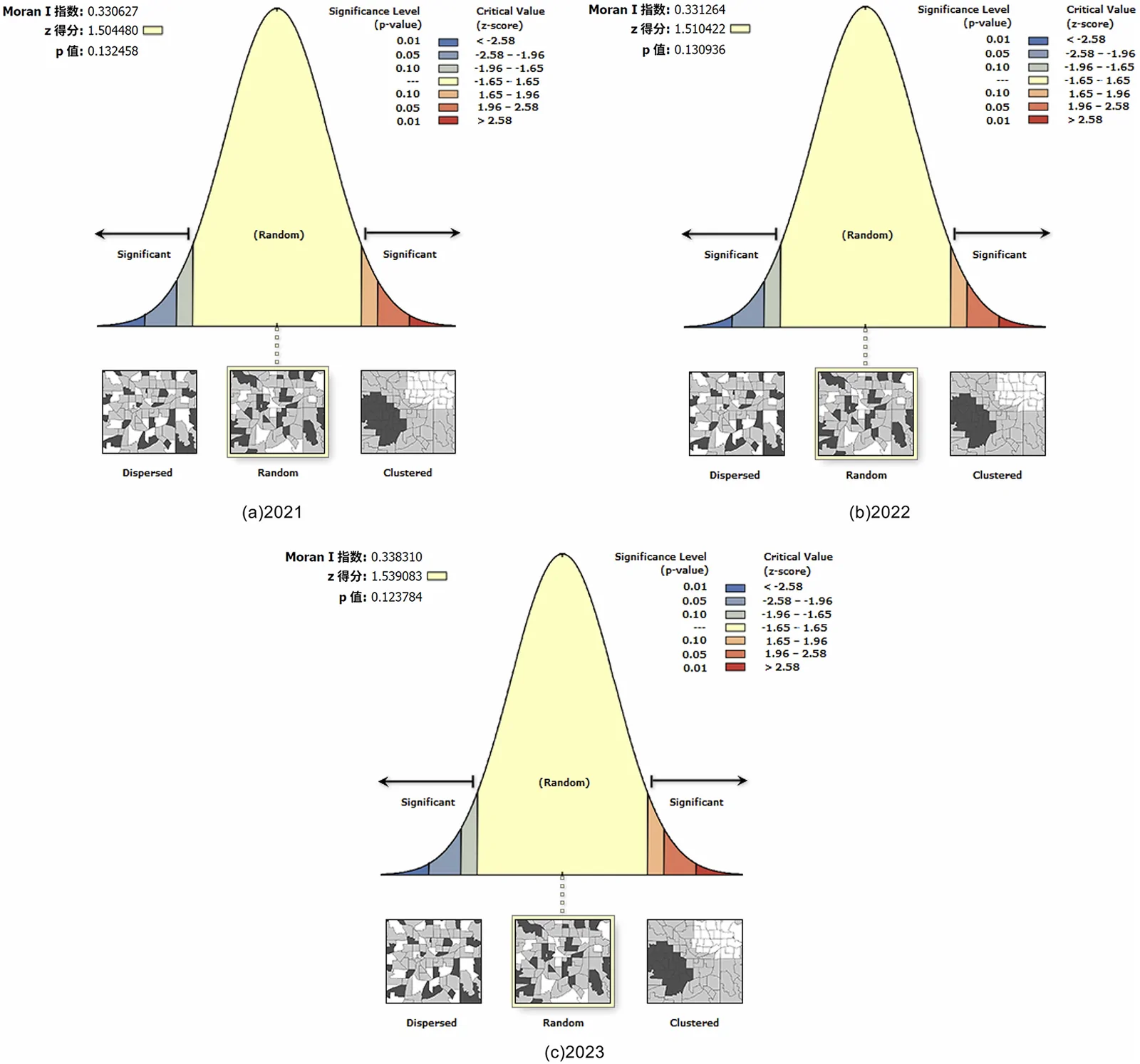
Global Moran's I normal distribution diagnostic plots of RECE from 2021 to 2023. **(a)** Global Moran's I normal distribution diagnostic plots of RECE in 2021. **(b)** Global Moran's I normal distribution diagnostic plots of RECE in 2022. **(c)** Global Moran's I normal distribution diagnostic plots of RECE in 2023.

Additionally, to investigate whether residential electricity carbon emissions (RECE) exhibit spatial dependence—and consequently evaluate the possible influence of spatial spillover effects on model estimates—this research employed the Global Moran's *I* index to assess spatial autocorrelation across the 146 valid samples. The spatial weight matrix was formulated utilizing the Queen's adjacent-edge and adjacent-vertex adjacency principles integrated within the ArcGIS Spatial Statistics module, which delineates residential area segments that share borders or geographic vertices as spatially proximate entities. In contrast to the distance threshold method or the k-nearest neighbors approach, the Queen adjacency rule eliminates the necessity for the manual establishment of subjective thresholds. Moreover, neighboring residential zones are more prone to produce direct spatial spillover effects owing to factors like microclimate interactions, communal amenities, and pedestrian movement; consequently, this principle is more applicable for defining physical spatial adjacency dynamics at the urban residential level. In accordance with the aforementioned weight configurations, this research performed global spatial autocorrelation analyses on the RECE of 146 sampled residential zones for the years 2021, 2022, and 2023, respectively. The numerical statistical findings are illustrated in Table 5, while the comprehensive normal distribution curves for Moran's *I* test across the 3 years are depicted in Figures 3a–c. The test outcomes indicate that the global Moran's *I* index for the years 2021 to 2023 has consistently maintained within the interval of 0.330 to 0.339, demonstrating a marginally positive value. Nonetheless, the *Z*-scores for each of the 3 years fell outside the required threshold of 1.96, with associated *P*-values exceeding 0.05, indicating that the statistical analysis failed to meet the 5% significance criterion. The findings suggest that the RECE values for residential area samples in the research region did not demonstrate notable geographical clustering or spillover effects; hence, the samples may be regarded as fulfilling the assumption of spatial independence. As a result, incorporating a spatial weight matrix is unnecessary when utilizing the random forest model in future analyses, offering adequate data-level rationale for this method.

**Table 5:** Global Moran's *I* test results of RECE from 2021 to 2023.

Year	Global Moran's *I*	*Z*-score	*P*-value	Spatial distribution feature judgment
2021	0.3306	1.5045	0.1325	No significant global spatial clustering; the distribution tends to be random
2022	0.3313	1.5104	0.1310	No significant global spatial clustering; the distribution tends to be random
2023	0.3383	1.5391	0.1238	No significant global spatial clustering; the distribution tends to be random

### 3.2 Characteristics of BEF

**Figure 4:**
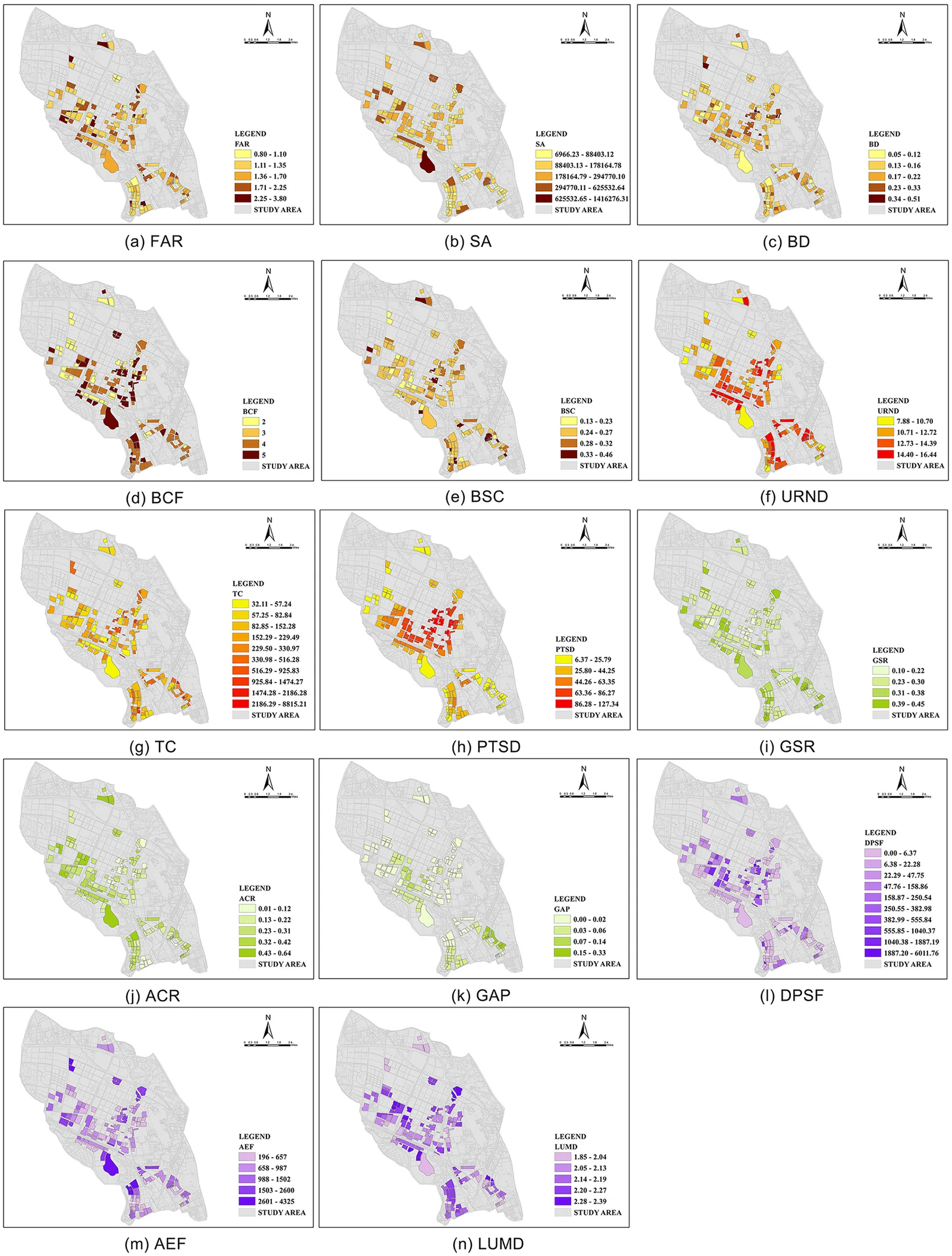
Quantitative spatial distribution map of built environment factors. **(a)** Quantitative spatial distribution map of FAR. **(b)** Quantitative spatial distribution map of SA. **(c)** Quantitative spatial distribution map of BD. **(d)** Quantitative spatial distribution map of BCF. **(e)** Quantitative spatial distribution map of BSC. **(f)** Quantitative spatial distribution map of URND. **(g)** Quantitative spatial distribution map of TC. **(h)** Quantitative spatial distribution map of PTSD. **(i)** Quantitative spatial distribution map of GER. **(j)** Quantitative spatial distribution map of ACR. **(k)** Quantitative spatial distribution map of GAP. **(l)** Quantitative spatial distribution map of DPSF. **(m)** Quantitative spatial distribution map of AEF. **(n)** Quantitative spatial distribution map of LUMD.

This study examines the spatial differentiation patterns of the built environment in residential communities within the study area by integrating the spatial distribution results of four categories of built environment sub-indicators, as illustrated in Figures 4a–n, across four dimensions: land use intensity, transportation layout, ecological environment, and public amenities. The land use intensity, as depicted in Figures 4a–e, indicates that the Floor Area Ratio (FAR) and Land Area (SA) are predominantly elevated in the northern region and diminished in the southern region. The BD indicator exhibits elevated values in the central and southern regions, while the eastern, northern, and western sectors display comparatively lower levels. Likewise, building layout indices such as Building Density (BD) exhibit a gradient pattern, with the central area surpassing the southern area, which in turn exceeds the northern area. This spatial pattern illustrates the temporal characteristic of residential land growth in the city, which is progressively extending southward from the central urban area, with the emphasis of land resource allocation transitioning from the core area to the southern districts as urban construction advances. In the transportation infrastructure dimension (Figures 4f–h), a dual-cluster pattern is evident in the central and southern regions: URND demonstrates dual-core agglomeration in the north and south; TC exhibits significant agglomeration in the south and a dispersed distribution in the central and northern areas; PTSD focuses on the central urban area as its core, with diminishing intensity toward the periphery, while a secondary agglomeration node develops in the southern district. In the ecological environment dimension (Figures 4i–k), the spatial distribution approaches general equilibrium, with elevated values of GSR and ACR uniformly distributed across the territory; only GAP shows spatial differences, being greater in the south and lower in the north. The Public Services Dimension (Figures 4l–n) reveals notable regional differences; the DPSF is predominantly concentrated in the central metropolitan area, but supporting resources are limited in the northern and southern districts. In contrast, the spatial distribution of AEF and LUMD is comparatively uniform. An examination of the spatial distributions across the four indicator categories indicates that the built environment in the research area exhibits considerable spatial disparities in resource allocation. It has established a spatial configuration characterized by a dual-core agglomeration comprising the central main city and the southern new district. The fundamental disparities in the built environment are crucial factors contributing to the north-south divergence and corridor-based concentration of residential electricity consumption and carbon emissions previously mentioned, offering empirical evidence at the built environment level for further examination of the mechanisms influencing spatial carbon emission patterns.

Residential zones within the research area exhibit considerable disparities in land-use intensity, transportation infrastructure, ecological conditions, and public services, with a dual-center configuration characterized by concentrations in the central and southern region.

### 3.3 Gray correlation analysis

**Figure 5:**
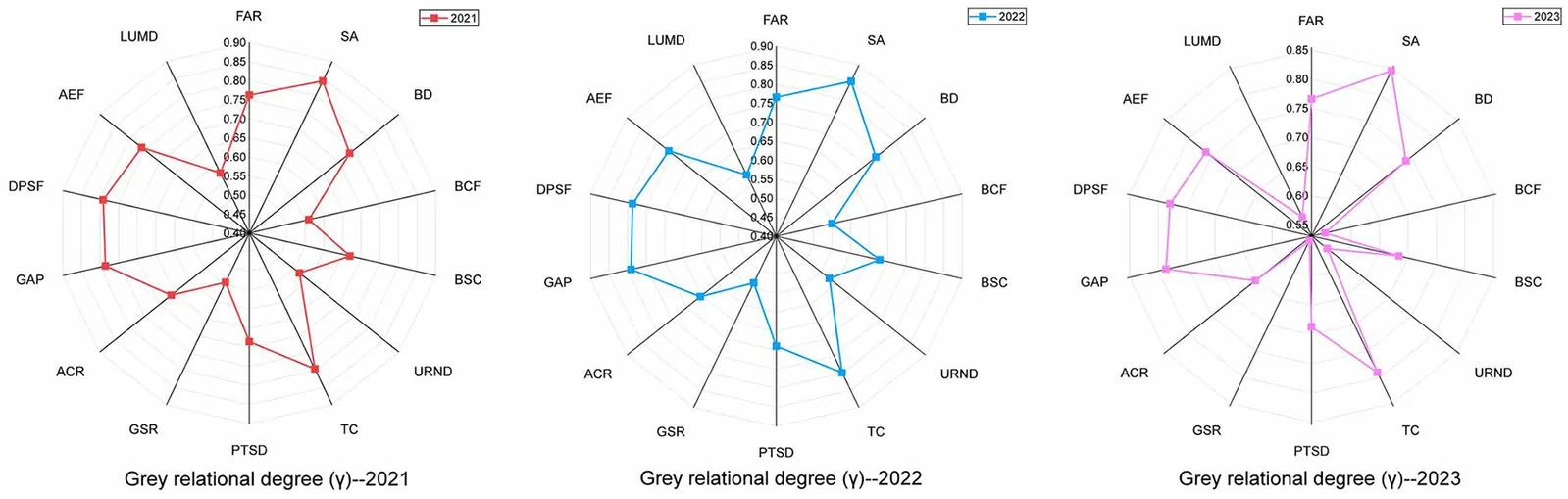
Grey correlation analysis of BEF and RECE from 2021 to 2023.

This research initially utilized gray correlation analysis to evaluate the correlation characteristics between building environmental factors and residential electricity carbon emissions. The findings indicate (Figure 5) that the gray correlation coefficients for each influencing factor were predominantly consistent from 2021 to 2023, with slight variations across the years, suggesting that the correlation framework between the influencing factors and the target variable demonstrated significant stability throughout the study duration. The gray correlation coefficients over the three-year duration varied between 0.53 and 0.85 in total. Every factor showed a varying level of association with carbon emissions, albeit with differing intensities. Considering the remarkable uniformity of the findings over the 3 years, the ensuing discourse predominantly emphasizes the analytical results for 2023. The findings from the 2023 gray correlation analysis reveal that SA exhibits the highest correlation coefficient, 0.846, indicating a strong alignment between the spatial dimensions of residential zones and fluctuations in residential electricity carbon emissions. An increase in SA is a crucial metric of residential area growth, generally correlating with increases in floor space, population density, and energy consumption, thereby reinforcing its link to fluctuations in carbon emissions. The fourteen factors of the built environment can be categorized into three tiers according to their correlation strength. The initial level consists of elements exhibiting strong association, each possessing a correlation coefficient exceeding 0.73. This level encompasses seven elements—SA, TC, GAP, DPSF, FAR, AEF, and BD—that address various aspects, including land-use scale, traffic arrangement, green area configuration, development density, and facility accessibility. This suggests a robust synergistic connection between residential electricity carbon emissions and the community's overall spatial layout and functional arrangement. Among these factors, spatial scale (SA) has the highest position with an association coefficient of 0.846, affirming the essential influence of community development scale on total energy demand; the increase in floor area and population size directly propels the inelastic rise in electricity consumption. It is important to note that while FAR and BD serve as metrics for land-use intensity, their correlation values differ slightly −0.766 and 0.737, respectively—indicating that high-density development influences electricity-associated carbon emissions in two distinct ways. The association coefficients for the three variables associated with resident mobility and public services—TC, DPSF, and AEF—were all more than 0.76. This implies that the accessibility of transportation in residential communities and the quality of service may indirectly impact residential power consumption by altering behavioral trends, including travel frequency, activity range, and duration of residence. Although this avenue is frequently underestimated in urban carbon emissions studies, these findings suggest that its significance must not be disregarded. The proportion of green space (GAP) exhibited a correlation coefficient of 0.786, placing it near the apex of the second tier. Its robust interplay with carbon emissions can be realized through microclimatic regulation methods—such as the cooling and humidifying benefits of green areas and the enhancement of building thermal environments—which indirectly reduce energy use for air conditioning. The secondary level comprises elements with modest correlations, fluctuating between 0.60 and 0.70, such as PTSD, BSC, and ACR. Elements within this category exhibit specific similarities in their variability, indicating meso- and micro-scale components of the built environment, including distinct building designs and the arrangement of transportation infrastructure. Although their interaction with carbon emissions is not as pronounced as that of macro-scale spatial structural elements, their correlation coefficients remain within a reasonable range, suggesting that these variables' influence on carbon emissions from electricity use may be contingent or indirect. The Building Form Factor (BSC) affects energy consumption in buildings by altering the shape coefficient and the heat-exchange area of the outer envelope; nonetheless, this influence is tempered by finer architectural design factors such as orientation and window-to-wall ratio, which diminish its overall impact at the neighborhood level. PTSD (public transportation stop density) and ACR (vegetation coverage rate) exert indirect effects on electricity consumption via travel substitution mechanisms and microclimate modulation routes. Given their extensive causal chains and multiple intermediary variables, the observed low correlation coefficients are consistent with anticipated outcomes. The tertiary level comprises low-correlation variables, each exhibiting correlation coefficients under 0.60, such as LUMD, URND, BCF, and GSR. The tenuous correlation between these elements and fluctuations in electricity-associated carbon emissions indicates that, within the framework devised in this research, factors like land-use diversity, urban roadway density, structural compactness, and solar exposure may not be the primary built environment determinants affecting residential electricity-related carbon emissions, or their impacts might be obscured by more dominant factors at the present spatial scale. The gray correlation analysis uncovered three principal findings: initially, numerous elements of the built environment demonstrate a degree of non-linear association with carbon emissions linked to household electricity, establishing a foundation for the later utilization of non-linear random forest models; Secondly, the potency of these correlations exhibits a diminishing hierarchy of ”spatial structure factors > building form factors > land use structure factors,“ implying that interventions at the spatial organization level may offer superior carbon-reduction capabilities compared to singular building designs; thirdly, the correlation strengths of each factor persisted consistently over the three-year span, signifying that these association trends are not attributable to random variations and demonstrate considerable temporal resilience. Nevertheless, gray correlation analysis is limited to assessing the extent of synchrony in variable trend changes; it fails to disentangle the independent impacts of individual factors while accounting for the influence of other variables, and it does not easily identify thresholds or inflection points in non-linear effects. Consequently, this research utilizes a random forest model to quantitatively evaluate the significance of influencing elements, overcoming the previously described constraints by building on the gray correlation analysis.

### 3.4 Random forest analysis

**Figure 6:**
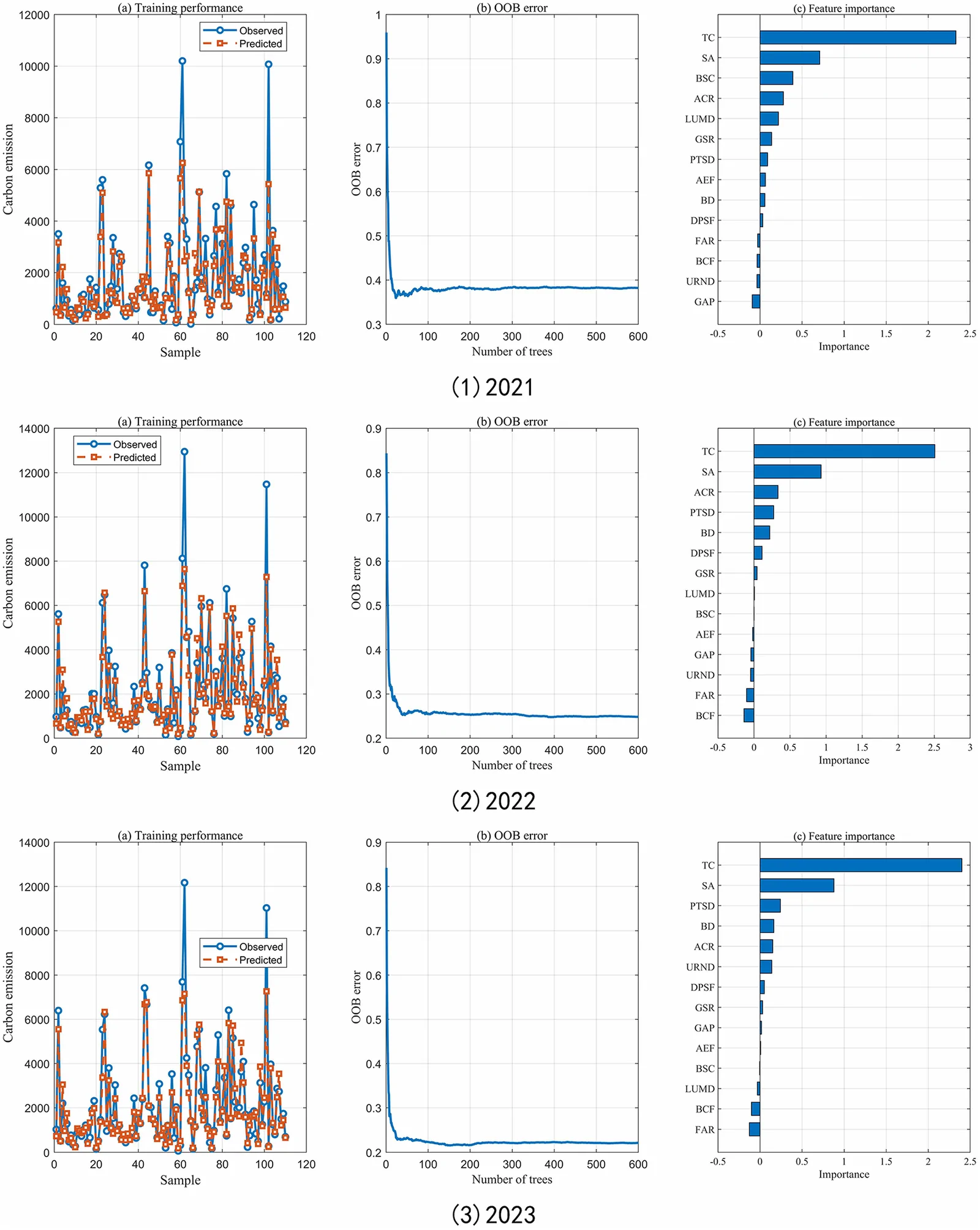
Fitting diagram of random forest model from 2021 to 2023. **(a)** Training performance of random forest model from 2021 to 2023. **(b)** OOB error of random forest model from 2021 to 2023. **(c)** Feature importance of random forest model from 2021 to 2023.

The research independently constructed Random Forest models utilizing data from 2021, 2022, and 2023 to elucidate the yearly fluctuation trends and non-linear attributes of the influence of building environmental factors on residential electricity carbon emissions. The research developed random forest regression models using MATLAB and employed consistent model parameter configurations for the 2021–2023 data to ensure comparability of model outcomes across years. The outcomes of the random forest models are illustrated in Figure 6. The models exhibited commendable prediction precision and consistent generalization capability throughout the study duration, suggesting that the chosen building environmental factors can proficiently elucidate the geographical variability of residential electricity carbon emissions. In 2021, the R^2^ for the training set was 0.842, for the test set it was 0.796, and the OOB Score was 0.598; in 2022, these figures were 0.862, 0.826, and 0.675, respectively; and in 2023, they were 0.875, 0.816, and 0.698, respectively. The R^2^ for the test set was consistently above 0.79 annually, and the disparity in performance between the training and test sets was negligible, suggesting that the model demonstrates excellent predictive reliability and robust generalization capabilities, with no notable overfitting detected. Moreover, the model's test set accuracy has remained stable over the years, reinforcing the applicability and dependability of the Random Forest model in elucidating the correlation between building environmental factors and residential electricity carbon emissions.

An analysis of the fitting outcomes for both the training and testing datasets (Figure 6a) indicates that, throughout the 3-year duration, the model's forecasts predominantly mirrored the variation trends of actual residential electricity carbon emissions. The precision of the fitting improved in the low- to medium-carbon-emission spectra, and the forecasted curves showed strong concordance with the empirical data. Despite minor discrepancies in a limited number of high-carbon-emission residential zones, the aggregate forecast error was within an acceptable range, exhibiting no significant systematic overestimation or underestimation. This suggests that the Random Forest model can proficiently uncover the non-linear association between attributes of the built environment and residential electricity carbon emissions. The OOBScore trend curve (Figure 6b) illustrates that in the early training phase, the error diminished swiftly with the augmentation of decision trees. As the quantity of decision trees approached 200–300, the error progressively leveled off, with only slight variations thereafter, signifying that the model had achieved stability and convergence with the existing parameter configurations. This indicates that increasing the number of decision trees would not markedly improve model performance, and that the model did not exhibit clear signs of overfitting. The results of feature importance (Figure 6c) demonstrate that the overarching trend of primary influencing factors has remained largely consistent from 2021 to 2023, with only slight annual fluctuations in the significance of specific indicators. Over the three-year span, TC consistently ranked highest in significance and contributed markedly more than other factors, establishing it as the principal determinant of the geographical variation in residential electricity carbon emissions. Transportation conditions consistently and significantly affect residential electricity carbon emissions: location accessibility and transportation options influence residents' daily travel decisions regarding public and non-motorized transport, as well as the habitual electricity use of personal electric vehicles and auxiliary heating devices. By impacting carbon emissions associated with household electricity consumption from the energy demand perspective, TC significantly enhances the model's efficacy; SA has consistently held substantial significance; the extent of land utilization directly influences the total capacity for households and permanent residents within a residential community. Disparities in population magnitude profoundly influence the fluctuations in a community's total power usage, distinctly demonstrating the essential regulating function that the spatial development framework of residential zones has on residents' energy consumption behaviors. The contribution of each indicator demonstrates considerable annual and periodic variations: in 2021, BSC had a relatively high impact, as building form—through its influence on building surface area and heat exchange—was a key factor affecting residential electricity carbon emissions; in 2022, the importance of ACR increased, as tree coverage influenced residential electricity carbon emissions by improving the neighborhood's microenvironment; in 2023, the importance of both PTSD and BD rose simultaneously; public transportation station density reduced reliance on private motorized travel by improving residents' travel efficiency and access to public services, thereby lowering transportation-related indirect energy consumption and electricity-related carbon emissions; Building density, on the other hand, reduces energy consumption per unit area by adjusting land-use intensity, increasing mutual shading between buildings, and reducing thermal loss through building envelopes; however, its energy-saving effects are constrained by building height and layout, resulting in a relatively limited overall impact within the study area. The synergistic and distinct influences of these two elements, along with the non-linear repercussions of the annual increase in household consumption, led to the cumulative effect of these indicators becoming increasingly evident in 2023. Conversely, metrics concerning floor area ratio and green space have persistently held minimal significance, suggesting that, within the parameters of this study's sample and spatial extent, these built environment elements are improbable to substantially influence residents' fundamental electricity usage and fail to adequately elucidate the spatial disparities in carbon emissions across residential zones, whether in a linear or non-linear context. The 3-year Random Forest model exhibited remarkable explanatory power and predictive precision, with the coefficient of determination on the test set consistently exceeding 0.79 and the OOB Score indicating solid stability and generalization capability. The hierarchy of significance among fundamental building environmental factors remained largely unchanged, indicating that the process by which the built environment affects residential electricity carbon emissions was relatively stable over the study period. These findings further validate that non-linear ensemble learning techniques are particularly effective and offer valuable insights into the intricate interconnections between the urban built environment and residential electricity carbon emissions.

## 4 Discussion

### 4.1 Comparison with existing methods

Based on the RECE measurement method proposed in this study, the electricity consumption of RB is calculated through information such as user names, electricity usage locations, electricity consumption, and voltage in AMI data, and then the RECE is calculated. Due to the accurate positioning, high timeliness and high coverage of AMI data, it can well make up for the shortcomings of traditional data collection that consume a lot of human and material resources, and overcome the problems of low data accuracy, poor timeliness, lack of representativeness and lack of authenticity in traditional methods (40). Compared with the traditional RECE measurement methods, this study proposes a RECE measurement method framework based on AMI data, which has the advantages of strong representativeness, accurate scale and wide scope. In 2024, the spatial allocation model was used to study urban construction land carbon emissions (41). Compared with the RECE measurement method framework based on AMI data proposed in this paper, this study can achieve the accuracy of RECE at the block level and greatly improve the timeliness, representativeness and realism. The remaining studies, based on the statistics of energy consumption and population in the statistical yearbook, were used to estimate the RECE (42). Questionnaire survey data, and energy consumption simulation data all have the same problem (43). Therefore, compared with traditional carbon emission estimation methods, this method has the advantages of strong representativeness, accurate scale and wide range.

### 4.2 Comparison with previous studies and underlying mechanisms

#### 4.2.1 Comparison with previous studies

This study demonstrates that built environment characteristics significantly influence household carbon emissions from energy consumption, with notable variations in the significance of these factors. Throughout the study period, transportation compactness (TC) and land area (SA) consistently demonstrated significant importance and were pivotal factors in regional variations in carbon emissions from residential energy use (Figure 6). This finding aligns with the conclusions of prior studies about the impact of transport accessibility and urban spatial organization on home energy usage. Prior studies consistently indicate that effective transport accessibility can reduce travel distances, increase the proportion of public transport and non-motorized travel, and decrease residents' dependence on motorized transport, thereby reducing energy consumption and carbon emissions (20).

In contrast to certain studies that highlight the predominant influence of building density, floor area ratio, and architectural form (44), our research determined that transportation-related variables are considerably more relevant than those pertaining to building design. This disparity may arise from variations in the study subjects and spatial surroundings. Most current research focuses on evaluations of central urban regions in megacities, where internal transportation conditions and public service levels are relatively uniform, leaving building density as the principal source of variation. Conversely, Wuqing District is situated in the peripheral new town region of Tianjin, comprising historical urban centers, urban-rural transition zones, and recently established residential neighborhoods. Marked differences exist across areas in transportation accessibility and public service availability; thus, the impact of location and transportation conditions on inhabitants' daily activities and energy consumption patterns is more pronounced.

This study demonstrated that the land area (SA) regularly has a substantial role in explaining overall carbon emissions. This indicates that neighborhood size not only mirrors the extent of land development but also affects total power consumption by influencing population density and household numbers. This discovery rectifies a deficiency in current research, which has predominantly focused on morphological attributes while largely overlooking the impact of neighborhood size, and offers novel empirical evidence to understand the mechanisms that govern residential energy consumption in suburban new towns.

#### 4.2.2 Underlying mechanisms of key factors

**Figure 7:**
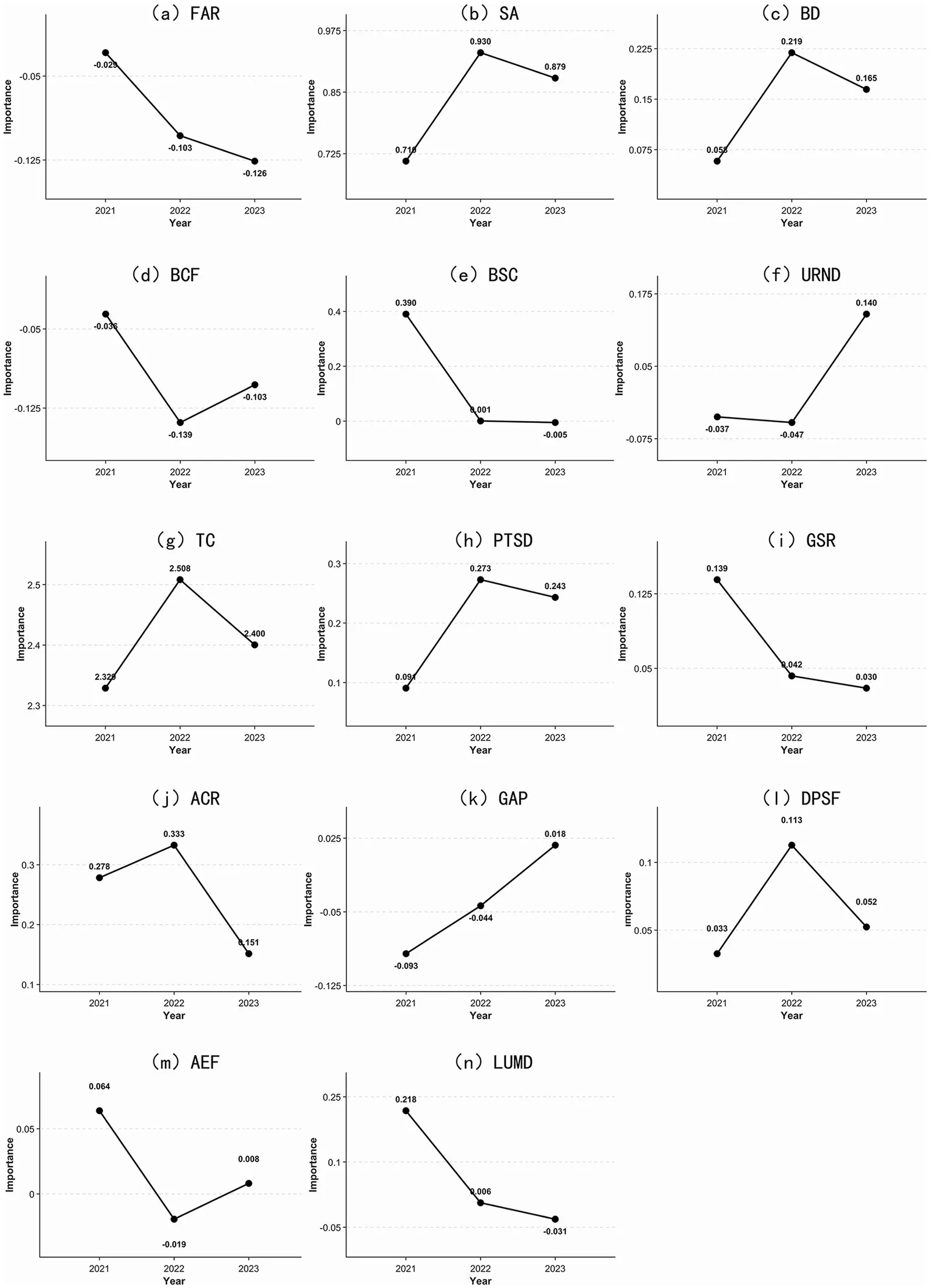
Trend of changes in the importance of built environment factors on residential electricity carbon emissions in 2021, 2022, and 2023. **(a)** Trend of changes in the importance of FAR on residential electricity carbon emissions in 2021, 2022, and 2023. **(b)** Trend of changes in the importance of SA on residential electricity carbon emissions in 2021, 2022, and 2023. **(c)** Trend of changes in the importance of BD on residential electricity carbon emissions in 2021, 2022, and 2023. **(d)** Trend of changes in the importance of BCF on residential electricity carbon emissions in 2021, 2022, and 2023. **(e)** Trend of changes in the importance of BSC on residential electricity carbon emissions in 2021, 2022, and 2023. **(f)** Trend of changes in the importance of URND on residential electricity carbon emissions in 2021, 2022, and 2023. **(g)** Trend of changes in the importance of TC on residential electricity carbon emissions in 2021, 2022, and 2023. **(h)** Trend of changes in the importance of PTSD on residential electricity carbon emissions in 2021, 2022, and 2023. **(i)** Trend of changes in the importance of GER on residential electricity carbon emissions in 2021, 2022, and 2023. **(j)** Trend of changes in the importance of ACR on residential electricity carbon emissions in 2021, 2022, and 2023. **(k)** Trend of changes in the importance of GAP on residential electricity carbon emissions in 2021, 2022, and 2023. **(l)** Trend of changes in the importance of DPSF on residential electricity carbon emissions in 2021, 2022, and 2023. **(m)** Trend of changes in the importance of AEF on residential electricity carbon emissions in 2021, 2022, and 2023. **(n)** Trend of changes in the importance of LUMD on residential electricity carbon emissions in 2021, 2022, and 2023.

**Figure 8:**
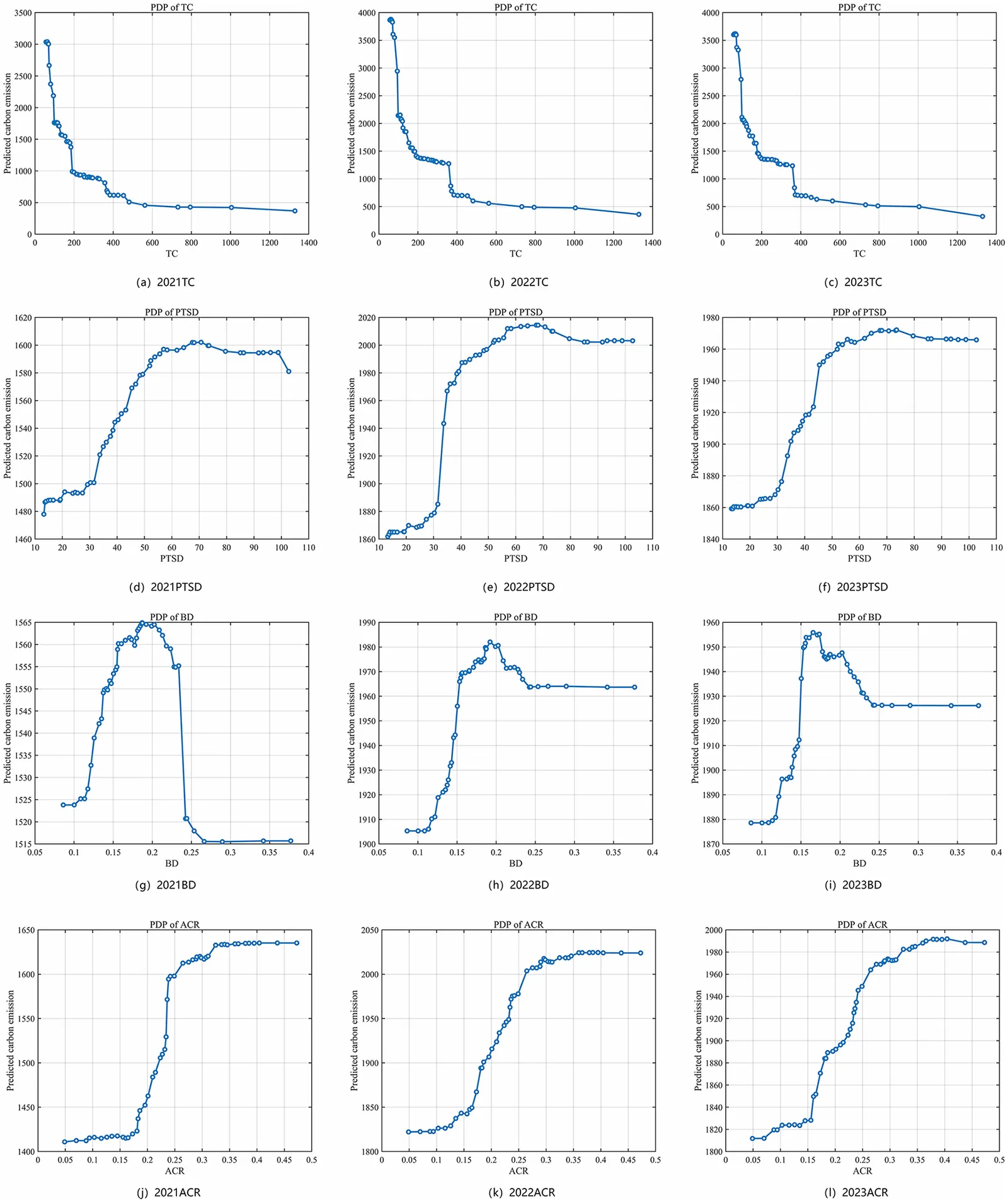
PDP analysis of key factors, 2021 to 2023. **(a)** PDP analysis of TC, 2021. **(b)** PDP analysis of TC, 2022. **(c)** PDP analysis of TC, 2023. **(d)** PDP analysis of PTSD, 2021. **(e)** PDP analysis of PTSD, 2022. **(f)** PDP analysis of PTSD, 2023. **(g)** PDP analysis of BD, 2021. **(h)** PDP analysis of BD, 2022. **(i)** PDP analysis of BD, 2023. **(j)** PDP analysis of ACR, 2021. **(k)** PDP analysis of ACR, 2022. **(l)** PDP analysis of ACR, 2023.

Utilizing the alterations in the significance of diverse indicators from 2021 to 2023 as identified by the Random Forest model (Figure 7), we identified consistently significant factors and employed PDP analysis to investigate the pathways through which critical building environmental factors affect residential electricity carbon emissions (Figure 8).

Transportation Compactness (TC) exhibits a substantial negative correlation with household electricity usage, suggesting that areas with enhanced public transit accessibility generally experience reduced carbon emissions from electricity consumption. This phenomenon can be attributed to sophisticated road infrastructure and enhanced access to amenities, which optimize residents' travel efficiency, mitigate traffic congestion, reduce unnecessary travel time, and improve access to public services, thereby lowering specific travel-associated indirect energy consumption (20). Moreover, areas with elevated accessibility generally exhibit a more cohesive urban functional layout that caters to the everyday needs of nearby residents, thereby fostering a more compact, low-carbon way of living. Public transportation stop density (PTSD) exerts a notable non-linear influence on carbon emissions. When PTSD is under 60, carbon emissions escalate as stop density amplifies. This is probably due to the underdevelopment of the public transportation system; new stops are mostly located in areas of population density and growing travel needs, while residents continue to rely heavily on personal vehicles. As a result, the escalation in emissions due to heightened travel demand surpasses the emission mitigation benefits of enhancements in public transit. Once PTSD surpasses 60, the public transportation system progressively evolves, and citizens' travel behaviors transition toward low-carbon alternatives. The influence of new stations on carbon emissions generally stabilizes, illustrating the diminishing marginal returns typical of public transit systems. Comparable research has indicated that the impact of public transportation on emission reduction is contingent not solely on the extent of infrastructure but is also affected by network efficiency and the travel behaviors of residents (45). Consequently, low-carbon transportation strategies ought to transition from merely augmenting the quantity of stations to refining the coverage and operational efficacy of public transit networks, thereby improving their capacity to replace private vehicle usage. Elements associated with architectural design demonstrate a more intricate pattern of impact. Research suggests that building density (BD) exhibits an inverted “*U*”-shaped correlation with carbon emissions resulting from power usage, while its total effect is minimal. At minimal building densities, the thermal barrier effect is not yet evident, and carbon emissions escalate with rising density, reaching a maximum when building density ranges from 0.15 to 0.25; subsequently, increased building density enhances the thermal shielding effect among structures, significantly lowering heating and cooling energy usage, which leads to a reduction in carbon emissions. Prior research has shown that augmenting building density can lead to energy conservation by decreasing the exposed surface area of building envelopes, enhancing shadowing among structures, and diminishing heat loss during winter (46, 47). This indicates that, in the research zone, construction density affects carbon emissions but is not a determining element. Unlike building density, the extent of tree coverage rate (ACR) exhibits a positive correlation with carbon emissions resulting from electricity usage. This discovery is somewhat at odds with earlier studies suggesting that green spaces alleviate the urban heat island phenomenon and diminish the need for building cooling (48, 49). One potential rationale is that areas rich in green spaces often coincide with higher-quality, refurbished residences, where residents generally have higher incomes, own more household appliances, and use more energy for comfort; this increased electricity use might offset the energy-saving benefits of green spaces. This implies that the connection between green areas and energy consumption could be affected by socioeconomic elements and the behaviors of residents, rather than adhering to a straightforward linear correlation.

#### 4.2.3 Implications for healthy and low-carbon community planning

This study's findings suggest that enhancing transportation infrastructure and adopting strategic spatial planning can reduce carbon emissions from home energy use and potentially improve public health. Prior studies indicate that walkable, public transit-oriented development can enhance physical activity among residents and mitigate health concerns, including obesity and cardiovascular disease (50, 51). Thus, enhancing road connections, upgrading public transportation systems, and developing comprehensive communities not only facilitate the low-carbon transition but also foster a healthier and more habitable urban environment.

This study revealed that the impacts of various built environment factors are significantly context-dependent, suggesting that low-carbon planning strategies should not merely replicate the experiences of other cities but should thoroughly consider differences in urban development stages, spatial structural characteristics, and resident behavioral patterns. In new towns and suburban regions like Wuqing District, prioritizing the optimization of transportation networks, enhancing the equitable distribution of public services, and fostering compact, moderate development may be more effective in achieving carbon-reduction objectives than merely increasing building density or the ratio of green space. This discovery offers specific empirical support for low-carbon community development and sustainable urban planning in China's emerging metropolitan regions.

### 4.3 Optimization strategy

This paper utilizes the national spatial planning framework (52) to formulate a comprehensive emissions reduction strategy across four dimensions: macro-scale spatial patterns, meso-scale built environments, micro-scale unit management, and long-term governance guidance. The strategy seeks to attain synchronized advancement in urban low-carbon building through planning, management, and public awareness, while reconciling the promotion of low-carbon transformation in residential communities with the improvement of living standards.

#### 4.3.1 Optimization of the comprehensive territorial spatial configuration

Enhancing the comprehensive spatial configuration of territory constitutes the macro-level basis for diminishing regional carbon emissions (53, 54). It can rectify the existing inequity in resource distribution within the built environment, alleviate spatial development disparities—such as the north-south divergence in carbon emissions from residential electricity usage and the concentration of high-emission zones—and attain equitable, low-carbon spatial development throughout the region (55).

Given the spatial attributes of the study area—particularly the dual-core resource concentration of the “central main city and southern new district” and the deficiency of supporting facilities in the north—spatial zoning management ought to be instituted. This involves coordinating the balanced allocation of public service facilities, moderately prioritizing the provision of transportation, commercial, and community amenities in high-carbon-emission areas in the north, mitigating the resource siphoning effect in core areas, and alleviating the north-south gradient in carbon emissions; Strictly enforce controls on territorial spatial development boundaries, strengthen constraints on development intensity along major transportation corridors, strictly regulate the disorderly expansion of residential communities along these corridors and development exceeding population carrying capacity, and prevent the sharp surge in electricity consumption caused by excessive population concentration; Implement differentiated zoning controls in high-carbon contiguous clusters, such as the southern section of Cuiheng Road and the central section of the North Canal, strictly limit the establishment of new high-end, large-unit, energy-intensive residential developments, and curb the spread of high-carbon spatial clusters.

#### 4.3.2 Stringent regulation of constructed environment metrics

The regulation of built environment indicators is essential for comprehensive land-use and spatial planning (56, 57). It is a crucial instrument for tackling the temporal variability of carbon emission drivers and effectively regulating carbon emission fluctuations within micro-communities (58). By utilizing sophisticated indicator control, the opportunity for passive emissions reduction in the built environment can be harnessed, hence improving the efficacy of low-carbon spatial governance (59, 60).

A revised classification control system is built based on the distinct driving patterns of factors revealed by the Random Forest model. Utilizing the enduring carbon-reduction benefits of locational accessibility, the configuration of public and non-motorized transportation systems throughout the region is perpetually refined to direct residents toward low-carbon travel and diminish regular electricity usage from household appliances. Simultaneously, stringent regulations are enforced on the developmental capacity of residential land and the population carrying capacity, while optimizing population distribution within established large residential communities to regulate electricity-related carbon emissions at the land-use level. By utilizing the energy-efficient characteristics of architectural form factors and building density, a systematic and compact layout in new residential communities is encouraged, with strict limitations on irregular and dispersed architectural designs, and a judicious management of building density parameters. Utilize the thermal barrier effect of buildings to reduce essential electricity consumption for heating and cooling; Leveraging the positive emission-reduction characteristics of tree coverage and diverse public facilities, we will optimize residential greening models, promote low-maintenance, low-energy-consumption mixed systems of trees, shrubs, and grasses, and strictly control excessive landscape lighting and the use of high-energy-consumption greening maintenance equipment; while adhering to the principle of meeting essential needs, strictly controlling the construction of high-end, redundant commercial and entertainment facilities on plots, retrofitting existing high-energy-consumption and low-efficiency supporting facilities, and avoiding increases in residential electricity demand caused by overly comprehensive supporting facilities; streamline specialized low-carbon control measures for inefficient indicators such as floor area ratio and standard green space requirements, concentrate resources on addressing core drivers, and enhance the precision of regulatory controls.

#### 4.3.3 Differentiated renewal management of community units

Community unit management functions as the micro-level execution mechanism for the revitalization of current land and spatial resources (61). By tackling fundamental issues such as the disparity in carbon emissions among communities and the varying developmental requirements of residential communities with high and low emissions (62), it can reconcile the dual objectives of reducing carbon emissions and enhancing living standards, thus creating a balanced, stratified low-carbon development framework for communities (63).

For communities like Huajun Jiayuan, characterized by elevated carbon emission intensity and a varied array of property types, create specific low-carbon management ledgers to facilitate targeted and exact emission reductions. Implement stringent regulations on electricity consumption for comfort in villas and upscale residences, while advocating for zoned metering in common areas and intelligent management of elevators and lighting in high-rise and mid-rise residential buildings to minimize unnecessary common-area electricity usage. For low-carbon, low-value communities characterized by uniform housing types and electricity consumption predominantly dictated by essential needs, we will transcend a limited carbon-control perspective. We will incorporate low-carbon quality enhancement into the old-community revitalization framework. By optimizing interior functions, incorporating low-carbon community amenities, and advocating for energy-efficient facilities, we will attain a balance between improved living standards and low-carbon development. For dispersed, low-value communities like Guanlan Garden, which face a potential increase in carbon emissions annually, it is essential to integrate low-carbon management into the community renewal process from the outset. Stringently control the rate of excessive enhancements to supporting infrastructure and the transition of business formats to premium categories to mitigate the chaotic increase in residential electricity consumption and protect the foundation of low-carbon development throughout the region.

#### 4.3.4 Long-term governance and awareness guidance for low-carbon initiatives

Long-term low-carbon governance is an essential soft component of the national territorial space governing framework (64, 65). By concentrating on residents' energy consumption patterns and long-term trends, it can reinforce the successes in balancing per-household electricity-related carbon emissions in residential communities, tackle the issue of concealed carbon increases due to escalating consumer demand, and attain synergistic empowerment through spatial regulation and behavioral direction (66, 67).

To enhance the trend of annual stability and uniformity in per-household electricity-related carbon emissions in the study area, a systematic low-carbon education and outreach program should be implemented. Utilizing neighborhood public service venues, initiatives such as science dissemination on energy-efficient power usage and the advocacy of low-carbon lifestyles should be implemented to systematically elevate citizens' understanding of low-carbon electricity consumption. This will assist residents in cultivating moderate, energy-efficient electricity consumption habits, mitigating excessive and high-energy-demand behaviors on the demand side, and preventing unintentional carbon increases resulting from enhancements in quality of life; Implement a dynamic monitoring system for residential electricity-related carbon emissions, concentrating on tracking fluctuations in energy consumption within low-value, middle-income communities and those undergoing renovation and improvement. Establish targeted low-carbon governance strategies to create a holistic low-carbon governance framework that incorporates geographic optimization, indicator-based regulation, and behavioral empowerment, thereby guaranteeing the sustained and gradual decrease of carbon emissions associated with household energy consumption.

### 4.4 Limitations and further improvements

This investigation presents certain constraints that ought to be rectified in further studies. Initially, the sample parameters and the duration are constrained. This research included only 146 residential communities within the central urban zone of Wuqing District, Tianjin, with energy usage statistics spanning 3 years from 2021 to 2023. The constraints of sample size and duration may somewhat hinder the representativeness and generalizability of the study's findings; specifically, their relevance to cities of varying sizes or distinct climate zones necessitates additional validation. Secondly, the resolution of built environment data needs enhancement. Certain indicators of the built environment are sourced from open-source mapping data and proprietary databases, which exhibit limitations regarding data precision, update regularity, and spatial resolution, potentially compromising the reliability of the indicator assessments. Third, not all sources of residents' total energy usage are included. This research examines carbon emissions from household electricity use and excludes winter heating and other energy consumption from the scope of analysis. In northern Chinese cities with frigid climates, like Tianjin, there exist notable disparities in energy types, efficiency of utilization, and supply frameworks across different heating methods—such as centralized heating, electric heating, and multi-energy hybrid heating—and their impact on residents' yearly total carbon emissions is significant; this research has yet to elaborate on these distinctions (68). Fourth, energy-related elements have not been integrated into the indicator framework. This research could not integrate energy-related variables—such as regional power supply composition, renewable energy adoption rates, and community energy infrastructure—into the model framework due to constraints in obtaining comprehensive energy system data. In the North China Power Grid area, which includes Tianjin, the significant reliance on thermal power generation and specific electricity distribution practices may affect the actual carbon emissions from household electricity use; nonetheless, the national average emission factors used in this research do not adequately capture these regional variations (69). Fifth, there is a lack of variables related to residents' socioeconomic traits and energy-use habits. This research omitted individual-level variables such as household income, household size, appliance ownership, and inhabitants' energy consumption preferences due to constraints in accessing micro-level survey data. In areas characterized by expansive living quarters and elevated environmental standards, the enhanced built environment is often associated with higher incomes, more spacious residences, and a broader array of household devices; the rise in carbon emissions from electricity use may result from the interplay between the built environment and inhabitants' lifestyles (70). Consequently, the elements of the built environment identified in this research and their respective impacts predominantly reflect statistical associations within the limitations of the current variable framework; their causal explanatory capacity requires additional validation using micro-level individual-level data. The previously described limits arise chiefly from objective constraints on data accessibility and the study's scope; they also delineate a clear direction for forthcoming research to advance in domains such as data augmentation, scale enhancement, and mechanism analysis. Initially, enhance the integration of multi-source, high-accuracy data and extend the observation period. Subsequent investigations may enhance the incorporation of high-resolution remote sensing photography, Internet of Things (IoT) monitoring apparatus, and community-based energy consumption tracking systems to amass extensive longitudinal data on the constructed environment and carbon emissions associated with power. This would strengthen the reliability of model parameter estimations and augment the temporal extrapolation potential of study findings. Secondly, implement an all-encompassing carbon emissions tracking system that encompasses various heating methods and numerous energy consumption categories. In the future, artificial intelligence algorithms may be integrated with IoT sensing technologies—such as intelligent electricity and thermal meters—to systematically collect data on communal heating systems, heat energy sources, and seasonal energy usage trends. This would facilitate the development of a home carbon emissions accounting system that integrates various energy consumption processes, such as electricity and heating, thus improving the accuracy of yearly carbon emissions assessments for residential communities in colder climates. Third, integrate variables from the energy supply sector to enhance investigations into the mechanics of carbon emission generation from a ”supply-demand“ coordination viewpoint. In the future, information from the energy supply sector—including regional power grid emission metrics, the allocation of renewable energy installations, and the operational characteristics of community energy facilities—may be further consolidated. Integrating the energy supply framework into the analytical model will enhance the understanding of how variations in electricity generation techniques influence carbon emissions from residential electricity use, thus overcoming the existing analytical constraint of concentrating exclusively on the spatial environmental aspect. Fourth, integrate socioeconomic and behavioral microdata to develop a comprehensive framework for identifying influential factors. Prospective studies may integrate surveys of residents' socioeconomic characteristics, documentation of household energy usage patterns, and minute-by-minute energy consumption data from smart meters to create a collaborative analytical framework connecting ”built environment—socioeconomic characteristics—energy consumption patterns. This would clearly differentiate between spatial structural influences and resident behavioral influences, enhance the precision of identifying determinants of carbon emissions, and furnish more precise, focused evidence for tailored low-carbon planning strategies. Fifth, broaden cross-regional comparison studies to validate the universality of the influencing mechanisms. Subsequent research may utilize this analytical framework across diverse climate zones, varying city sizes, and distinct urban development paradigms for comparative analysis. This would systematically evaluate the consistency and variability of the identified influencing mechanisms across contexts, thereby improving the external validity of the research findings and providing theoretical support with broader relevance for the planning and design of low-carbon residential communities suited to local circumstances.

## 5 Conclusions

This research examines 146 residential communities in Wuqing District, Tianjin, to systematically investigate the spatial variability of RECE from 2021 to 2023, develop a comprehensive set of built environment assessment metrics, and perform a fitting analysis utilizing a random forest model to elucidate the mechanisms through which building environmental factors affect residential electricity carbon emissions. The primary findings are as follows: (1) This research utilizes residential electricity carbon emissions data from 146 residential communities in Wuqing District between 2021 and 2023 to elucidate the stratification and differentiation patterns of carbon emissions from both temporal and spatial viewpoints, while extracting the varied driving mechanisms of the constructed environment. Over time, overall carbon emissions shifted from rapid growth to a stable state, characterized by a deceleration in emission intensity and a gradual convergence of per-household emissions, indicating the saturation of residential electricity use and the rising uniformity of energy consumption. At a macro level, spatial characteristics of the built environment—like land utilization, geographic placement, and public amenities—propelled by population density, primarily influenced the spatial distribution of overall carbon emissions; nonetheless, they failed to account for the spatial discrepancies in carbon emission intensity and emissions per household. The distinct response traits of these three categories of indicators validate the practicality of examining the driving forces behind residential electricity-related carbon emissions through the lens of the built environment, offering empirical support for enhanced low-carbon management and targeted emission reduction in residential zones. (2) The constructed environment within the research zone displays spatial disparities across four dimensions—land utilization, transit, ecology, and public amenities—ultimately creating a dual-core configuration consisting of the central urban zone and the southern new district; urban land development has transitioned southward from the central urban zone, with transportation and public service resources predominantly located in the central and southern areas, whereas ecological metrics show a relatively uniform distribution throughout the region. The spatial distribution of the constructed environment establishes the essential parameters for the geographical disparity in residential carbon emissions and serves as a reference point for examining the factors influencing these emissions. (3) Utilizing gray correlation and random forest analysis, a notable non-linear association was identified between built environment variables in the research area and residential electricity carbon emissions. Empirical findings suggest that transportation compactness (TC) and land area (SA) are fundamental long-term factors influencing the geographical variation in carbon emissions from residential energy use. As urban infrastructure enhances and the quality of life for people increases annually, the influential factors of building form factor (BSC), tree coverage rate (ACR), public transportation stop density (PTSD), and building density (BD) demonstrate a sequential, fluctuating upward trajectory. Every indication establishes unique energy consumption transmission routes according to its specific characteristics: minimizing building surface area modulates heat transfer, consequently impacting electricity usage; extensive green spaces lead to heightened electricity demand for comfort-driven devices; public transit systems shape residents‘ commuting behaviors, subsequently influencing carbon emissions associated with residential electricity; and concentrated development mitigates the escalation of energy consumption by altering land-use intensity and providing thermal insulation benefits. Conversely, metrics such as floor area ratio and green space distribution are constrained by the uniformity of residential offerings in the research zone, resulting in a persistently low explanatory power for fluctuations in residential electricity carbon emissions. These results further validate that the Random Forest model is exceptionally adept at delineating the intricate, multifaceted interactions of carbon emission pathways inside the constructed environment. This research underscores the pivotal influence of transportation networks, the intensity of land development, and spatial configuration on residential electricity carbon emissions. It offers a scientific foundation and a pragmatic strategic direction for regional low-carbon initiatives, enhancing urban infrastructure, indirectly benefiting inhabitants' health, and achieving carbon peak and neutrality objectives.

## Data Availability

The raw data supporting the conclusions of this article will be made available by the authors, without undue reservation.
